# Precise Sizing and Collision Detection of Functional Nanoparticles by Deep Learning Empowered Plasmonic Microscopy

**DOI:** 10.1002/advs.202407432

**Published:** 2025-01-10

**Authors:** Jingan Wang, Yi Sun, Yuting Yang, Cheng Zhang, Weiqiang Zheng, Chen Wang, Wei Zhang, Lianqun Zhou, Hui Yu, Jinghong Li

**Affiliations:** ^1^ School of Biomedical Engineering Shanghai Jiao Tong University Shanghai 200030 China; ^2^ Department of Chemistry Center for BioAnalytical Chemistry Key Laboratory of Bioorganic Phosphorus Chemistry and Chemical Biology Tsinghua University Beijing 100084 China; ^3^ Chinese Academy of Sciences Key Laboratory of Urban Pollutant Conversion Department of Environmental Science and Engineering University of Science and Technology of China Hefei 230026 China; ^4^ School of Sensing Science and Engineering School of Electronic Information and Electrical Engineering Shanghai Jiao Tong University Shanghai 200030 China; ^5^ Suzhou Institute of Biomedical Engineering and Technology Chinese Academy of Sciences Suzhou 215163 China

**Keywords:** collision, deep learning, microscopy, nanoparticle, plasmonic

## Abstract

Single nanoparticle analysis is crucial for various applications in biology, materials, and energy. However, precisely profiling and monitoring weakly scattering nanoparticles remains challenging. Here, it is demonstrated that deep learning‐empowered plasmonic microscopy (Deep‐SM) enables precise sizing and collision detection of functional chemical and biological nanoparticles. Image sequences are recorded by the state‐of‐the‐art plasmonic microscopy during single nanoparticle collision onto the sensor surface. Deep‐SM can enhance signal detection and suppresses noise by leveraging spatio‐temporal correlations of the unique signal and noise characteristics in plasmonic microscopy image sequences. Deep‐SM can provide significant scattering signal enhancement and noise reduction in dynamic imaging of biological nanoparticles as small as 10 nm, as well as the collision detection of metallic nanoparticle electrochemistry and quantum coupling with plasmonic microscopy. The high sensitivity and simplicity make this approach promising for routine use in nanoparticle analysis across diverse scientific fields.

## Graphical abstract

An alternative approach to enable the precise sizing and collision detection of nanoparticles is demonstrated using deep learning empowered plasmonic microscopy (Deep‐SM). The exceptional sensitivity enables direct analysis of extracellular particles as small as 10 nm and probing the quantum coupling between a 20 nm gold nanoparticle and a thin film.

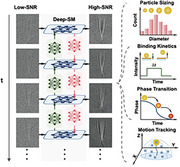

## 1 Introduction

Nanoparticles are extensively utilized across various scientific and industrial domains. In medical imaging, they serve as essential cargos and labels for drug delivery, imaging, and diagnostics.^[^ ^1^ ^]^ In nanomedicine, they can be employed for magnetic therapy by exploiting magnetothermal and magneto‐mechanical effects.^[^ ^2^ ^]^ In energy storage, conversion, and transmission systems, nanoparticles like nanosized lithium iron phosphate and other nanostructured materials are employed in lithium‐ion batteries to enhance their energy density and cycling stability.^[^ ^3^ ^]^ There is also growing concern about the potential health risks associated with the consumption of particles such as microplastics from environmental pollution and contamination.^[^ ^4^ ^]^ Therefore, nanoparticle analysis is crucial to understanding the fundamental mechanism and to the development of new technologies in these applications.^[^ ^5^ ^]^ Rare nanoparticles with unique characteristics and functions that deviate from the majority are usually key to the discovery of new materials and phenomena.^[^ ^6^ ^]^ Electron microscopy and atomic force microscopy (AFM) provide excellent image contrast and high spatial resolution for nanoparticle characterization, however, they lack the capability for real‐time detection.^[^ ^7^ ^]^ Conventional approaches such as dynamic light scattering (DLS) are inadequate for addressing the intrinsic heterogeneity and fail to reveal important rare events due to their low resolution and limited mass accuracy.^[^ ^8^ ^]^ This underscores the pressing need for the development of advanced single nanoparticle analysis techniques.

Scatter‐based microscopy techniques utilize the intrinsic scattering characteristics of nanoscale objects, enabling label‐free imaging without the need for fluorescent markers.^[^ ^9^ ^]^ For instance, interferometric scattering microscopy (iSCAT) has set a benchmark for high sensitivity, enabling detection at the single‐molecule level.^[^ ^10^ ^]^ Surface plasmon resonance microscopy (SPRM) is an alternative approach for nanoparticle analysis with unique capability in observing molecular interaction and interfacial electrochemical processes.^[^ ^11^ ^]^ A primary challenge in SPRM imaging technique, however, is the inherently weak scattering signal of nanoparticles, often overwhelmed by substantial background noise.^[^ ^12^ ^]^ In an ideal plasmonic microscope, subtracting a static background image should reduce the major noise in the single nanoparticle image to shot noise, which arises from the quantum nature of light.^[^ ^13^ ^]^ Existing SPRM techniques typically work with nanoparticles larger than 50 nm and further improving the sensitivity could extend its applications such as to detect binding kinetics and electrochemical processes of nanoparticles of only a few nanometers. Current advancements in SPRM aim to address these challenges, driving applications toward smaller entities, such as single exosomes and macromolecules.^[^ ^14^ ^]^ However, these approaches typically require extreme updates on the imaging sensor, light source, and optical system, and high sensitivity was achieved at strong illumination and reduced temporal resolution.^[^ ^15^ ^]^ In practice, it also requires ultrahigh stability of the optical system and working environment to reach the shot‐noise limit, especially as the moving average strategy decreases the temporal resolution. This hampers its wide use in analyzing biological nanoparticles or ultrafast processes.

The application of deep learning has emerged as a transformative approach to enhance the imaging quality of microscopes, with significant advancements such as super‐resolution imaging, noise reduction, virtual image staining, etc.^[^ ^16^ ^]^ Although these approaches achieve notable improvements without increasing the complexity of the optical setup, many fail to address the fundamental challenges associated with shot noise in scatter‐based imaging.^[^ ^17^ ^]^ Given the inherently random and unpredictable nature of shot noise, the underlying design principles and rationale behind these methods are not always clearly articulated. Here, we present Deep‐SM, an innovative deep‐learning framework for plasmonic microscopy, capable of achieving precise nanoparticle sizing and collision detection across diverse particle types. This framework incorporates a spatio‐temporal feature learning network—comprising a 2D Residual U‐Net (ResUNet) and a bi‐directional convolutional long short‐term memory (Bi‐ConvLSTM) network—to synergistically capture both spatial and temporal correlations in plasmonic image sequences recorded during nanoparticle collisions. By learning the unique spatial patterns and correlative spatiotemporal noise characteristics specific to plasmonic microscopy, Deep‐SM significantly enhances scattering signal detection and reduces noise without altering the optical setup or compromising spatial and temporal resolution. We showcase the effectiveness of Deep‐SM by demonstrating its high‐sensitivity detection of single nanoparticles, tracking of extracellular nanoparticles, and precise monitoring of collision electrochemistry in metallic nanoparticles. This capability broadens the potential applications of SPRM, making Deep‐SM a promising tool for advanced nanoparticle analysis in various scientific and industrial domains.

## 2 Results and Discussion

### 2.1 Deep Learning Empowered Plasmonic Microscopy

**Figure 1:**
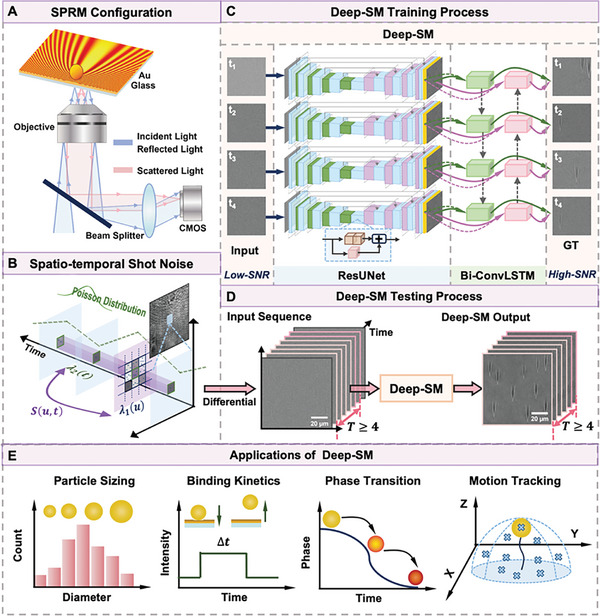
Schematic of The Deep Learning‐empowered Plasmonic Microscopy (Deep‐SM). A) Schematic of SPRM imaging system. B) Spatio‐temporal correlations of shot noise in SPRM image sequences explained by Cox process. C) Deep‐SM comprised a sequential structure of ResUNet and Bi‐ConvLSTM modules. The noisy SPRM image sequence was first fed into ResUNet to extract spatial features of particles and shot noise, and then further denoising was achieved by Bi‐ConvLSTM through temporal information exploration. D) The shot noise‐contaminated SPRM sequence (*N* ≥ 4) with low SNR could be enhanced to a high‐SNR sequence by Deep‐SM. E) Applications of Deep‐SM in SPRM imaging system.

The spatiotemporal feature learning network worked with the typical SPRM system without increasing the complexity of the optical setup. Basically, surface plasmon resonance was stimulated with the Kretschmann configuration by parallel light illumination at the resonance angle (**Figure** **1**A).^[^ ^18^ ^]^ This configuration is widely utilized for exciting surface plasmon polariton (SPP) waves and their interactions with the sample at the metal‐dielectric interface.^[^ ^19^ ^]^ In the SPRM system, the far field image (*I_Raw_*) records the interference signal between the scattering field (*E*_*S*_) and the reflected field (*E*_*R*_), producing a characteristic “parabolic” pattern as,

(1) $$I_{\mathrm{Raw}}={\left|E_{R}+E_{S}\right|}^{2}={\left|E_{R}\right|}^{2}+{\left|E_{S}\right|}^{2}+{E_{R}}^{*}E_{S}+E_{R}{E_{S}}^{*}$$

where *E*_S_ results from the interaction of the SPP field with the samples. After subtracting the background image (|*E*_R_|^2^), the major noise source in the SPRM image sequences was supposed to be shot noise, arising from the variation in the time that photons reach the detector. Specifically, the noise at each pixel varies follows the Poisson distribution over time, and the noise in each image also varies over space stochastically (Figure 1B). Such a process could be described as the Cox process, also known as the doubly stochastic Poisson process^[^ ^20^ ^]^ (Note S1 and Figure S1, Supporting Information). The shot noise distribution *λ*(*u*, *t*) in SPRM image sequences could be expressed as a multiplicative decomposition equation,^[^ ^21^ ^]^

(2) $$\lambda \;\left(u,t\right)=\lambda_{1}\;\left(u\right)\lambda_{2}\left(t\right)S\left(u,t\right),\;\mathrm{ES}\;\left(u,t\right)=1,\;\left(u,t\right)\in R^{2}\times Z$$

where *λ*_*1*_(*u*) is the noise distribution in the spatial domain, and *λ*_*2*_(*t*) refers to the noise variation over time. *S*(*u*, *t*) represents the spatio‐temporal process with unit mean, given by the surrogate of spatio‐temporal covariates of shot noise. This suggests that by studying the 2D distribution of shot noise in time and space, it is possible to further improve the sensitivity limited by shot noise.

We thus constructed a spatio‐temporal feature learning network named Deep‐SM to enhance the sensitivity of SPRM. It is composed of a series of ResUNet and Bi‐ConvLSTM networks (Figure 1C; Note S2, Supporting Information). The rationale for designing Deep‐SM includes the two following aspects. First, the interference mechanism in plasmonic microscopy provides spatial information that contributes to the overall image sequences, which typically exhibit “parabolic” patterns. We have taken this into account by incorporating the ResUNet structure into the construction of Deep‐SM. ResUNet integrates Convolution Neural Network based U‐Net and Residual Network (ResNet), which is superior in capturing spatial features of SPRM images. The structure of ResUNet is adapted from a four‐layer U‐Net by replacing the double convolution block with a residual convolutional block for each layer in U‐Net (Figure S2A and Table S1, Supporting Information). U‐Net features a four‐layer encoder and decoder structure, which preserves the information on parabolic patterns in SPRM images through down‐sampling and up‐sampling processes.^[^ ^22^ ^]^ The residual structure is introduced by adding the input to the output of the double convolution block in U‐Net for each layer. Since the shape of the SPRM pattern is relatively simple, the residual block can address the gradient disappearing and explosion issues in the traditional U‐Net training process.

Second, Deep‐SM was specifically designed to address the unique characteristics of shot noise in SPRM image sequences. These images contain shot noise with distinct spatiotemporal correlations which follow the Cox process. Therefore, we sequentially integrated Bi‐ConvLSTM with ResUNet to construct Deep‐SM. Bi‐ConvLSTM compromises two separate ConvLSTM modules, with one extracting features from the forward input time series and the other from the reverse processing (Figure S2B, Supporting Information). ConvLSTM replaces the fully connected layer in LSTM with the convolution layer by replacing the Hadamard product with the convolution operation.^[^ ^23^ ^]^ It is capable of capturing temporal correlations while preserving the spatial characteristics of both shot noise and the signal. By leveraging the inherent properties of shot noise, Deep‐SM provides a more robust and precise denoising approach. To capture temporal features, the input to Deep‐SM should consist of image sequences containing at least four SPRM frames. The number of frames can be increased depending on the quality of input images (Figure 1D). Additionally, the size of the input images should be a multiple of 16, as the ResUNet architecture includes four layers, each requiring a down‐sampling operation.

By leveraging the spatio‐temporal correlation of shot noise and signal features, Deep‐SM could enhance the sensitivity of the SPRM system without the need for modifications to the imaging configuration or compromising spatial and temporal resolution. Therefore, Deep‐SM has the potential for broad applications, including particle sizing, detection of binding kinetics, phase transitions, and motion tracking (Figure 1E).

### 2.2 Precise Sizing of Single Nanoparticles

**Figure 2:**
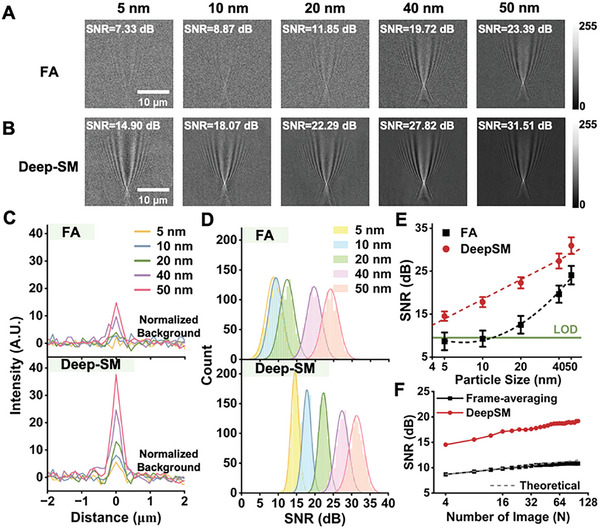
Deep‐SM Test on GNPs with Different Sizes. (A and B) FA A) and Deep‐SM results B) on 1000 GNPs at each different diameter (5, 10, 20, 40, and 50 nm) and their corresponding SNR. Scale bar: 10 µm. C) SNR analysis of GNPs with different sizes detected by FA (top) and Deep‐SM (bottom). D) Fitted Gaussian distribution of SNR of GNPs with different sizes detected by FA (top) and Deep‐SM (bottom). Error bars indicate mean ± SD. E) Calibration curves of 1000 GNPs with each different sizes detected by FA (black) and Deep‐SM (red). Error bars indicate mean ± SD. F)The SNR analysis of frame‐averaging (black) and Deep‐SM (red) results on 1000 GNPs at 5 nm using different numbers of input frames.

To evaluate the performance of Deep‐SM for single particle analysis, we applied it to both simulated (Figure S3, Supporting Information) and experimental SPRM image sequences (Figure S4, Supporting Information). We first applied it to detecting gold nanoparticles (GNPs) with different diameters (5, 10, 20, 40, and 50 nm). The number of input frames for Deep‐SM model could be increased based on the quality of the SPRM sequences. We compared the results obtained using Deep‐SM with those by averaging four consecutive frames (FA, **Figure** **2**A,B). The signal‐to‐noise ratio (SNR) was calculated from the normalized line profile of each single particle by subtracting the mean background noise and then dividing by the standard deviation (SD) of the background noise (shown in dB, Note S3, Supporting Information). The intensity of GNPs detected by Deep‐SM corresponded well with the particle size, with obviously larger SNR than FA detection (Figure 2C).

Besides, we statistically quantified the SNR of 1000 particles at each size, revealing a good fit with the Gaussian distribution function (Figure 2D). The distribution curves of 5, 10, and 20 nm GNPs were distinctly discernible in Deep‐SM results, whereas they exhibited considerable overlap in FA results. The GNPs were characterized in advance using DLS, which confirmed their good monodispersity (Figure S5, Supporting Information). Although aggregates may have been present in the samples, their concentrations were insufficient to detect a significant number of particles within the field of view. We thus believe that the results correspond to single particles. The SNR calibration curves for GNPs of different sizes thus indicated that Deep‐SM could improve the SNR up to approximately twofold and presented a stable increasing relationship with the logarithm of particle diameter (Figure 2E). For tiny nanoparticles at 5 and 10 nm which are below the limit of detection (9.54 dB), the scattering light was overwhelmed by the noise, resulting in low SNR of 8.68 ± 2.07 dB and 9.29 ± 1.88 dB in FA detection. In contrast, Deep‐SM detection substantially increased the SNR to 14.53 ± 1.09 dB and 17.80 ± 1.18 dB, respectively. We also evaluated the enhancing stability of Deep‐SM by comparing the intensity of several GNPs at 5 nm in Deep‐SM results, which corresponded well with the detected intensity in the Ground Truth (GT) acquired by an average of 100 adjacent frames (Figure S6, Supporting Information). We further demonstrated the performance of Deep‐SM using varying numbers of input frames (N ≥ 4) by analyzing the SNR change of 1000 GNPs at 5 nm in both multi‐frame averaging and Deep‐SM results (Figure 2F). The SNR enhancement achieved by the multi‐frame averaging approach improves proportionally with the square root of the number of averaged frames ($\sqrt{N}$).^[^ ^24^ ^]^ Our findings indicated that the enhancement capability of Deep‐SM remained stable and consistent with multi‐frame averaging results as the number of input frames increased. As previously stated, a key feature of Deep‐SM is its ability to explore the spatio‐temporal characteristics of shot noise. Therefore, its performance may decline if shot noise is not the dominant noise source in SPRM image sequences—potentially due to factors such as mechanical vibrations or systematic drift.

### 2.3 Extracellular Particle Analysis

**Figure 3:**
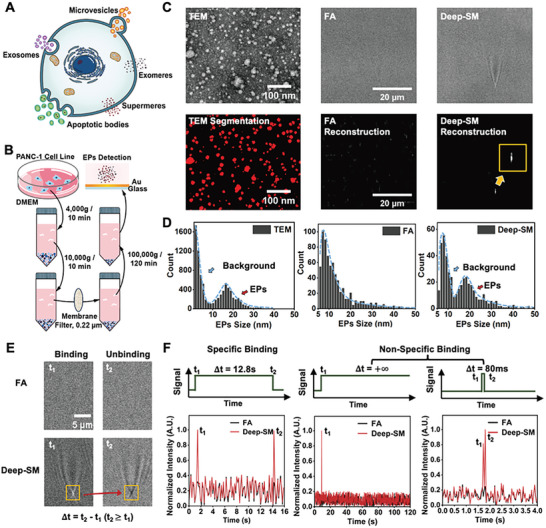
Precise Sizing of EPs (≤ 30 nm) Collected From PANC‐1 Human Pancreatic Cancer Cell Line. A) Categories of EPs in cells. B) Schematic diagram of isolation procedure of EPs. C) TEM, FA, and Deep‐SM figures of EPs (top line) and their corresponding segmentation and reconstruction results (bottom line). Scale bar of TEM images: 100 nm; Scale bar of FA and Deep‐SM images: 20 µm. D) Size distribution of EPs measured by TEM, FA, and Deep‐SM detection of SPRM. E) Binding (white center, t_1_) and unbinding (black center, t_2_) events detected by FA and Deep‐SM. Bound lifetime Δt can be calculated by t_2_‐t_1_. F) A classic specific binding event, two types of non‐specific binding events of an EP, and their corresponding SNR change along time in FA (black) and Deep‐SM detection (red).

Extracellular particles (EPs) are membrane‐bound protein‐nucleic‐acid complexes that carry distinct proteo‐transcriptomic signatures, including exomeres, supemeres, and lipoproteins (**Figure** **3**A).^[^ ^25^ ^]^ EPs have been proven to be relevant to various types of cancer and contain a variety of molecular markers. The detection of EPs at the single‐particle level primarily relies on transmission electron microscopy (TEM) and AFM due to their small size.^[^ ^26^ ^]^ Our previous work has demonstrated the imaging and detection of single extracellular vesicles (EVs), such as exosomes, with the interferometric reconstruction of SPRM images. However, the sensitivity of this method proved insufficient for EPs analysis, even with frame averaging.^[^ ^24^, ^27^ ^]^ The low SNR in SPRM images of a single EP would result in reconstruction artifacts that were indistinguishable from true EPs. Deep‐SM offered an effective solution for precisely quantifying the size distribution of EPs and analyzing their binding and unbinding events. Here, the EPs were collected from PANC‐1 human pancreatic cancer cell line (sourced from ATCC) via multiple differential centrifugation steps (Figure 3B). We applied the interferometric reconstruction metric based on Richardson‐Lucy deconvolution (Note S4 and Figure S7, Supporting Information) on both FA and Deep‐SM results and compared them with TEM segmentation results (Figure 3C). The size distribution of EPs was determined using the calibration curve of polystyrene nanoparticles (PSNPs), with adjustments made to compensate for the refractive index differences between PSNPs and EPs (Note S5 and Figure S8, Supporting Information). The counting statistics from reconstructed Deep‐SM results revealed an EPs size distribution of ≈18.5 nm, which was in good accordance with the size distribution measured by TEM (≈17 nm) (Figure 3D). In contrast, EPs can hardly be distinguished from the background according to the distribution histogram of FA results.

Due to the exceptional sensitivity without compromising the temporal resolution, Deep‐SM enables the monitoring of dynamic processes such as the binding of EPs to CD63 aptamer. The modification process of the CD63 aptamer onto the Aurum (Au) film and the detailed experimental procedures are described in the Experimental Section (see Figure S9, Supporting Information). CD63 is a specific marker protein in EVs, playing a crucial role in various cellular processes and facilitating the sorting of cellular proteins in late endosomes and multi‐vesicles, promoting the formation of exosomes.^[^ ^28^ ^]^ The bounding lifetime (Δt) of EPs was calculated by the difference between the unbinding time (t_2_) and binding time (t_1_) (Figure 3E). Three distinct binding profiles were found in Deep‐SM results, similar to those reported for specific binding at the level of seconds, and nonspecific binding or Brownian motion (Figure 3F; Note S6 and Figure S10, Supporting Information). However, this was not distinguishable from FA results (Movie S1, Supporting Information).

### 2.4 Single Nanoparticle Electrochemistry by Collision Detection

**Figure 4:**
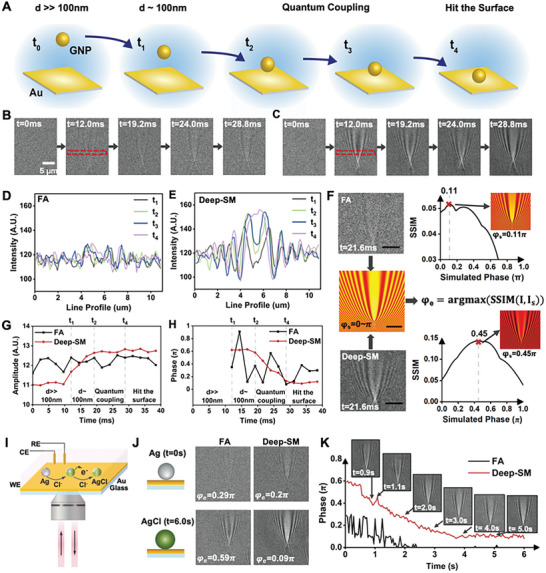
Rapid Phase Change Detection in Low‐SNR SPRM Images. A) Collision process of a single 20 nm GNP with bare Au film. The center of the parabolic “tail” turns from black to white after the entire collision process. (B and C) FA B) and Deep‐SM results C) at t_0_ = 0 ms (d⪢100 nm), t_1_ = 12.0 ms (d∼100 nm), t_2_ = 19.2 ms, t_3_ = 24.0 ms (quantum coupling), t_4_ = 28.8 ms (hit on the surface). Scale bar: 5 µm. D,E) Averaged Line profiles of a GNP within height of 15 pixels (dashed rectangular region) detected by FA D) and Deep‐SM E) during the collision process at t_1_ (black), t_2_ (green), t_3_ (blue), t_4_ (pink). F) Phase measurement metric based on SSIM algorithm. G,H) Amplitude G) and phase change H) during the whole collision process detected by FA (black) and Deep‐SM (red) results. I) Schematic of a single Ag to AgCl nanoparticle compositional evolution process detected by the SPRM system. J) FA and Deep‐SM results of a single Ag nanoparticle at t = 0 (top line) and a single AgCl nanoparticle at t = 6.0 s (bottom line), respectively. K) Phase value change during Ag to AgCl transformation process in FA (black) and Deep‐SM (red) detection and corresponding Deep‐SM images.

The Deep‐SM framework is readily applicable for various studies using SPRM, as no additional experimental processes are needed. To demonstrate it, we further applied it in the collision electrochemical assays. SPRM is well recognized as a powerful tool for monitoring collision electrochemistry at the single nanoparticle level, such as the quantum tunneling between nanoparticles and the electrode surface process. Nanoparticles can only be detected when they drop to within ≈100 nm of the Au surface (t_1_) (**Figure** **4**A). As nanoparticles approach the Au surface to ≈1 nm, quantum coupling takes the place of the classical electromagnetic coupling until they finally hit on the Au surface (t_2_ to t_4_).^[^ ^29^ ^]^

For the tunneling between GNPs and Au film, previous studies have been performed mainly on GNPs at 60 nm due to the detection limitation in sensitivity.^[^ ^29b^ ^]^ The Deep‐SM could be readily applicable to improve its accuracy in quantitative and dynamic monitoring of these processes. We quantified both the amplitude and phase change of 20 nm GNPs in the SPRM image sequences during its collision with the Au film with or without Deep‐SM enhancement. Phase changes (phase difference between the scattered field and the SPP field) could be clearly observed in the parabolic pattern (black‐center to white‐center) in Deep‐SM detection, while they were not discernible in FA results (Figure 4B,C). The averaged line profiles also turned out that the phase change of SPRM images by Deep‐SM was much clearer during the whole collision process than FA detection (Figure 4D,E). Besides, we measured the amplitude change during the collision process in 5  ×  5 area centered around the brightest point of the particle, and the phase change was determined by optimizing the structure similarity index measure (SSIM) between experimental and simulation images (Figure 4F; Note S7, Supporting Information). From the Deep‐SM results, it is clear that the amplitude of GNPs increased, and the phase remained unchanged as GNPs dropped from ≈100 to 1 nm near the bare Au film (t_1_ to t_2_) (Figure 4G,H). When quantum coupling occurred, the detected phase decreased to 0.13 π (white‐center) from 0.58 π (black‐center), while the amplitude remained unchanged. As GNPs finally hit the Au surface, both amplitude and phase were kept constant. In contrast, the variation of amplitude and phase could not be observed due to the limited sensitivity in FA detection. Thus, the Deep‐SM could extend the study of quantum tunneling to smaller metal nanoparticles or monitor tiny changes occurring within the gap between particle and film.

We also demonstrated another example of monitoring the oxidation process of single Argentum (Ag) nanoparticles. We used low illumination power at 2.3 mW cm^−2^ to mimic the situation with rapid detection using extremely short exposure time. In 20 mm potassium chloride (KCl) solution, by applying a voltage between Au film and a reference electrode in the solution, Ag nanoparticles were oxidized to silver chloride (AgCl) (Figure 4I). This induced a change in the phase from 0.59 π (black‐center) to 0.09 π (white‐center), while this phase transition could not be detected in FA images (Figure 4J; Movie S2, Supporting Information). With Deep‐SM, phase change during Ag to AgCl corresponded well with the simulation results in previous research (Figure 4K). The particle diameter also changed as Ag was oxidized to AgCl, which was reflected by the intensity change within the region of interest around the 5  ×  5 area around the brightest center. We further demonstrated the performance of Deep‐SM in rapid phase detection by setting the input frames as SPRM images with distinct phase values (Figure S11, Supporting Information). It turned out that Deep‐SM also worked efficiently in rapid phase change detection as well.

### 2.5 Nanoparticle Tracking and Localization at Nm‐Precision

**Figure 5:**
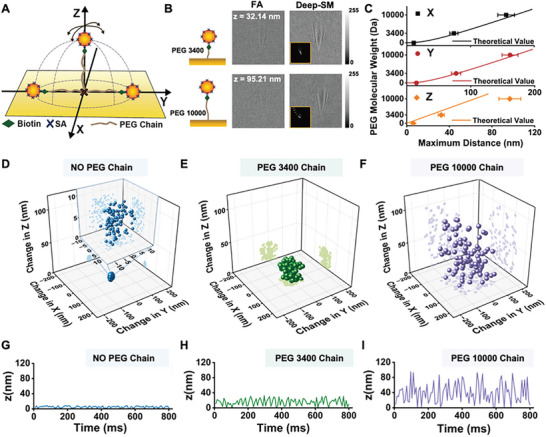
Brownian Motion Tracking of Stationary GNPs and GNPs Tethered to PEG chains. A) Schematic of PEG tethered GNPs. B) FA, Deep‐SM, and corresponding interferometric reconstruction results of a 50 nm GNP furthest from the Au film during motion. The reconstructed intensity was calculated by the average of 5  ×  5 area around the brightest center. C) The maximum motion distance of 400 GNPs in X, Y, and Z directions increases with PEG chain length and corresponding theoretical value. Error bars indicate mean ± SD. D–F) A typical 3D trajectory (X, Y, Z) of 50 nm GNP without PEG chain D), tethered to PEG 3400 chain E) and PEG 10 000 chain F). G–I) Trajectory of this GNP in Z direction without PEG chain G), tethered to PEG 3400 chain H) and PEG 10 000 chain I).

Nanoparticle tracking and localization enable continuous monitoring of single particles, such as biomarker monitoring based on particle mobility.^[^ ^30^ ^]^ Here, we demonstrated the performance of Deep‐SM in tracking the 3D Brownian motion (X, Y, Z) of 50 nm GNPs tethered to Polyethylene Glycol (PEG) linkers (Figure S12, Supporting Information). The GNPs were attached to PEG chains with a molecular weight of 3400 Da (PEG 3400) or 10 000 Da (PEG 10 000) by Streptavidin‐Biotin (SA‐Biotin) interaction, with the PEG chains anchored to the gold surface through Au─SH bonds. (**Figure** **5**A). We tested 400 GNPs with a diameter of 50 nm each linked to PEG 3400 chains and PEG 10 000 chains, and 400 stationary GNPs without any modification as the control group (Figure 5B). Then, we applied the interferometric reconstruction metric on both FA and Deep‐SM results and calculated the maximum motion distance of GNPs in X, Y, and Z directions. The localization precision based on Gaussian fitting in reconstructed Deep‐SM detection (Figure S13, Supporting Information), calculated by the SD of the trajectory of 400 stationary GNPs, was ≈3 nm in both X and Y directions, and ≈5 nm in Z direction. The moving distance of GNP centers in Z direction can be calculated by *I * = *I*_*0*_ *e* ^*−z/d*^, where *I* represents the intensity of the reconstructed image in 5  ×  5 area centered around the particle, *I*_*0*_ refers to the detected intensity when the particle is closest to the Au surface, and *d* denotes to the decay of the evanescent field (≈100 nm).^[^ ^31^ ^]^

In addition, we analyzed the maximum moving distance of GNPs functionalized with PEG 3400 and PEG 10 000, as well as that of the static GNPs in the control group. The maximum moving distance of nanoparticles is determined by two key factors: the diameter of the nanoparticles and the length of the tethered PEG chains. The theoretical value of the maximum moving distance of GNP centers in X and Y directions (*D* *x*_*max*_, *Dy*_*max*_) can be calculated by:^[^ ^31^ ^]^

(3) $$Dx_{\max},\;Dy_{\max}=2\times \sqrt{{\left(\frac{\mathrm{GNP}\;\mathrm{Diameter}}{2}+\mathrm{PEG}\;\mathrm{Chain}\;\mathrm{Length}\right)}^{2}-{\left(\frac{\mathrm{GNP}\;\mathrm{Diameter}}{2}\right)}^{2}}$$

which equals to 78.73 nm for PEG 3400 chains, and 170.08 nm for PEG 10 000 chains.^[^ ^32^ ^]^ The theoretical value of the maximum moving distance of GNP centers in Z direction (*D* *z*_*max*_) equals to the PEG chain length (Note S8, Supporting Information). We note that the evanescent wave nature of surface plasmons enables the highly sensitive detection of nanoparticle motion near the sensor surface. However, the localization accuracy in X and Y directions could decrease dramatically for particles located more than 50 nm above the sensor surface, due to the rapid decline in SNR caused by the exponential decay of evanescent waves in Z direction. This limitation highlights why plasmonic microscopy is generally more effective for detecting motions of nanoparticles close to the sensor surface. Therefore, we focused on analyzing the maximum displacement of GNPs in the X and Y directions within the data where Z ≤ 20 nm. In Deep‐SM results, the average Dx_max_ of stationary GNPs, GNPs tethered to PEG 3400 and PEG 10 000 chains near the Au surface (Z ≤ 20 nm) was 10.80 ± 1.21, 73.91 ± 6.42, and 155.92 ± 13.08 nm (Figure 5C). The average Dy_max_ was 15.16 ± 1.93, and 77.20 ± 7.25, 161.41 ± 12.46 nm. This is reasonable because the PEG chains could be coiled and difficult to stretch to full length. The difference in the motion range between a typical stationary GNP, a GNP tethered to PEG 3400 and PEG 10 000 chains could be clearly observed in their 3D and Z‐direction trajectory (Figure 5D–I). The recorded 3D trajectory of nanoparticles (Figure 5D–F) did not show semi‐spherical patterns, as the localization of particles in the X and Y directions was insufficiently precise at large Z‐values due to the reduced image contrast.

## 3 Conclusion

In the last decade, plasmonic sensors have advanced from ensemble detection to single‐particle analysis. One of the major challenges to further push the sensitivity limit lies in the inherent random noise which is previously thought to be the fundamental limit. We present the deep learning approach to overcome this barrier, achieving unprecedented sensitivity in detecting weakly scattering nanoparticles. This approach is readily applicable to data collected from well‐known surface plasmon resonance microscopy, enabling precise nanoparticle analysis down to 10 nm. This framework could achieve enhanced sensitivity without increasing the complexity of the optical setup or compromising temporal resolution, making it readily applicable for a range of applications such as nanoparticle counting, sizing, and tracking in biological samples at the nanometer scale. Additionally, it proves effective for studying dynamic processes, including molecular binding and electrochemical reactions. Given the unprecedented performance and simplicity of Deep‐SM, we anticipate its widespread application across various fields of nanoparticle analysis. Preliminary tests on images from other techniques, such as dark‐field microscopy and iSCAT microscopy (Figure S14, Supporting Information), suggest that this approach may also facilitate other scattering‐based imaging platforms.

## 4 Experimental Section

### SPRM System Configuration

The SPRM experiments were conducted using an inverted microscope (Olympus IX83) in the Kretschmann configuration, equipped with a high numerical aperture 60 × oil immersion objective (N.A. = 1.49). The sensor chips consisted of 2 mm BK7 glass substrates coated with 2 nm of chromium (Cr) and subsequently with 47 nm of gold (Au) film via magnetron‐sputtering technique. The incident light at a 655‐nm laser (MRL‐III‐655 L‐180 mW) was refocused and collimated, and it illuminates the chip being tested at an inclined angle (SPR angle) to minimize the reflected light intensity and maximize the scattering signal. The interference signal of reflected light and scattering light was then collected by a CMOS camera (Prime 95B; Photometrics) to capture the SPRM image sequences.

### SPRM Image Acquisition

Before the experiments, the Au chips were first cleaned with absolute ethyl alcohol and deionized water successively. To guarantee the smoothness of the chip surface, the chips were then fired with H_2_ flame after being dried by nitrogen. The chip was first modified with 1.156 g cm^−3^ polylysine from Sangon Biotech diluted with water at 1:10 vol/vol for ten minutes and then pipetted. The 50 nm gold nanoparticles purchased from Nanoportz diluted 1000 times in deionized water and diluted PBS from Beyotime at 1:10 vol/vol were then added to the polydimethylsiloxane (PDMS) attached to the modified chip. The landing process of the nanoparticles could be immediately observed. To acquire SPRM image sequences with different SNR and contrast, the current of the laser was set as 90, 100, 110, and 120 mA to illuminate the samples. The imaging area was made up of 500  ×  500 pixels, with a full acquiring full field of view (FOV) of ≈54 µm  ×  54 µm. The exposure time of CMOS was set to 2 ms, and 500 frames per second were recorded to guarantee that the dominant noise in differential images was shot noise. The single differential SPRM images, processed after background subtraction under the aforementioned imaging conditions, generally exhibited relatively low SNR, making it difficult to discern particles with parabolic‐tail patterns.

### Data Preparation and Network Training Parameters

Differential SPRM images were acquired by subtracting the background image from the raw SPRM image sequence. We trained Deep‐SM using 1000 sets of differential SPRM sequences, and the ratio of training data over validation data is 0.7 to 0.3. The nanoparticles remained stationary in half of the SPRM sequences, while the position of nanoparticles changed over time in the other half of the data. The training target of Deep‐SM was the average of 100 adjacent frames to acquire high‐SNR data. The training and validation data pairs were acquired under varying illuminance conditions and incident angles to generate SPRM image sequences with different noise levels and phase variations. The loss function used for training and evaluating Deep‐SM was the mean square error (MSE) between the Deep‐SM output and target,

(4) $$\begin{array}{ccc} \mathrm{MSE}\;\left(\mathrm{Deep}\mathrm{SM}\;\mathrm{Outp}\mathrm{ut},\;\mathrm{Targ}\mathrm{et}\right) & = & \frac{1}{N\cdot M}\;\sum_{n=1}^{N}\sum_{m=1}^{M}\\ & & {\left(\mathrm{Deep}\mathrm{SM}\;\mathrm{Outpu}t_{\mathrm{nm}}-\mathrm{Targe}t_{\mathrm{nm}}\right)}^{2} \end{array}$$

where N is the number of input frames to Deep‐SM, and M is the total pixel number of each frame. The additional training parameters include 1000 training epochs, with models saved every five epochs, and the final model selected based on the lowest validation loss. The initial learning rate of Deep‐SM was set to 10⁻⁴, with a learning rate decay factor of 0.5 applied every 50 epochs. We utilized the Adam optimizer, as it surpasses other optimizers in both convergence speed and loss function minimization. The minimum batch size for Deep‐SM training and validation was set to 32, with data pairs shuffled at each epoch to prevent overfitting of the Deep‐SM model. The model was implemented on a Linux‐based system using Pytorch, and training and testing of Deep‐SM were performed on the server with Intel Xeon Sliver 4116 CPU, 256GB RAM, and four Nvidia GeFore REX 2080 Ti graphic cards.

### Cell Cultivation

The PANC‐1 human pancreatic cancer cell line (sourced from ATCC) was utilized in this study for EPs collection. The cells were propagated in high‐glucose Dulbecco's modified Eagle's medium (DMEM) procured from Hyclone, supplemented with 10% extracellular‐vesicle‐free fetal bovine serum (EV‐free FBS) from SeraPro, and 1% penicillin‐streptomycin from Gibco. Cultivation was performed at 37 °C in a humidified incubator with 5% CO_2_, manufactured by Thermo Fisher Scientific. Cells were seeded in a T75 flask from Corning at 30% confluency and the culture medium was harvested after 48 h of growth, at which point the cells had attained 70–80% confluency. This collected culture medium was stored at 4 °C for 1 week.

### EPs Isolation and Specific Binding Assay

EPs were isolated via differential centrifugation. The harvested cell culture medium was initially centrifuged at 4000 g for 10 min to eliminate cells. The resultant supernatant was then centrifuged at 10 000 g for 10 min to discard cell debris. The processed medium was subsequently filtered using a 0.22 um membrane filter from Millipore. Lastly, the filtrate was subjected to ultracentrifugation at 100 000 g for 120 min. All the steps involved in the isolation process were conducted at 4 °C. The isolated EPs were preserved in the supernatant. To image the EPs specifically, the CD63 aptamer was anchored onto the surface. In brief, the sensor chamber was added 20 µL of Thiol‐PEG‐biotin at a concentration of 1 µm in 1x PBS for 10 min. Then the slide was rinsed three times with 1x PBS and incubated with 20 µL of 1 mg mL^−1^ streptavidin. Finally, 20 µL of 50 nm biotin‐aptamer was immitted and incubated for 4 h. A scramble aptamer and poly‐L‐Lysine were exploited as the control condition. All aptamer used in this work were synthesized and purified by GENEWIZ. The sequence of the CD63 ‐aptamer was: 5′‐ CAC CCC ACC TCG CTC CCG TGA CAC TAA TGC TAT TTT TTT TTT‐biotin‐3′. The sequence of the scrambled aptamer is: 5′‐ GCT ACC TCC CGA TAT TGA GGG CGC CCT CGT CTT TTT TTT TTT‐biotin‐3′ (Table S2, Supporting Information).

### Electrochemical Assay

The electrochemical experiments utilized a three‐electrode setup, with a gold chip serving as the working electrode, and an Ag/AgCl wire (Shanghai YueCi Electronic Technology Co., Ltd., China), and a Pt wire (Zhongke Keyou Technology Co., Ltd., China) as the reference and counter electrodes, respectively. The experiments focused on the impact of 70 nm silver nanoparticles, which were obtained from Qinkona New Materials Technology Center. To investigate their behavior under constant potential conditions, the Ag stock solution was appropriately diluted, and an 80 mm KCl solution from Macklin was introduced into the PDMS cell. The electrochemical cell was then supplemented with the silver nanoparticles at a potential of +0.15 V. The individual silver nanoparticles collided randomly, resulting in observable oxidation behaviors.

### GNPs Tethered to PEG Linkers

HS‐PEG‐Biotin with a molecular weight of 3400 Da, HS‐PEG‐Biotin with a molecular weight of 10 000 Da, and mPEG‐SH with a molecular weight of 500 Da from Aladdin were diluted to 1 µM. Then, the mixture HS‐PEG‐Biotin and mPEG‐SH was added to the holes of the PDMS at the ratio of 10:1. The chip was incubated overnight at room temperature, and the liquid in the hole was replaced by PBS at 1:10 vol/vol. Then, the 50 nm GNPs with a concentration of 10^10^ NPs/mL diluted × 100 were added to the holes of PDMS. After 20 min, the Brownian motion of GNPs tethered to the PEG linkers could be observed.

### Statistical Analysis

The data analysis was performed on MATLAB. The intensity and amplitude of nanoparticles were calculated within a 5  ×  5‐pixel area centered on the brightest point of each nanoparticle in both FA and Deep‐SM images. For the SNR analysis and maximum displacement analysis of nanoparticles, results are reported as means ± SD.

## Conflict of Interest

The authors declare no conflict of interest.

## Supporting information



Supporting Information

Supplemental Movie 1

Supplemental Movie 2

## Data Availability

The data that support the findings of this study are available from the corresponding author upon reasonable request.
