# Correction to “Precise Sizing and Collision Detection of Functional Nanoparticles by Deep Learning Empowered Plasmonic Microscopy”

**DOI:** 10.1002/advs.75592

**Published:** 2026-05-10

Jingan Wang^1^, Yi Sun^2,3^, Yuting Yang^4,^*, Cheng Zhang^4^, Weiqiang Zheng^1^, Chen Wang^1^, Wei Zhang^5^, Lianqun Zhou^5,^*, Hui Yu^1,^*, and Jinghong Li^2,*^, “Precise Sizing and Collision Detection of Functional Nanoparticles by Deep Learning Empowered Plasmonic Microscopy,” *Advanced Science* 12, no. 9 (2025): e07432, https://doi.org/10.1002/advs.202407432.

**This article corrects the following**:

https://doi.org/10.1002/advs.202407432

In the published article, we found that the image corresponding to the FA result at t = 24.0 ms in the **Figure 4B** was mistakenly replaced with the image of t = 28.8 ms during organizing the figures. The corrected Figure 4B is presented below.

**Corrected Figure 4B**:


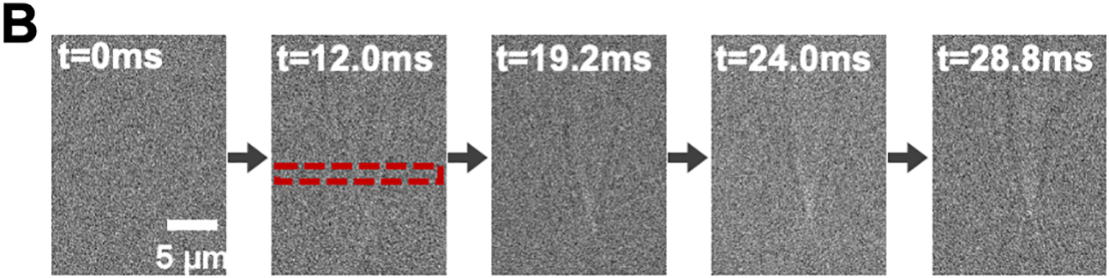


In the Supporting Information, the legend in **Figure S9** was inappropriately used. The labels for Biotin‐CD63 Aptamer and Thiol‐PEG‐biotin were inadvertently interchanged. The corrected Figure S9 is presented below.

**Corrected Figure S9**


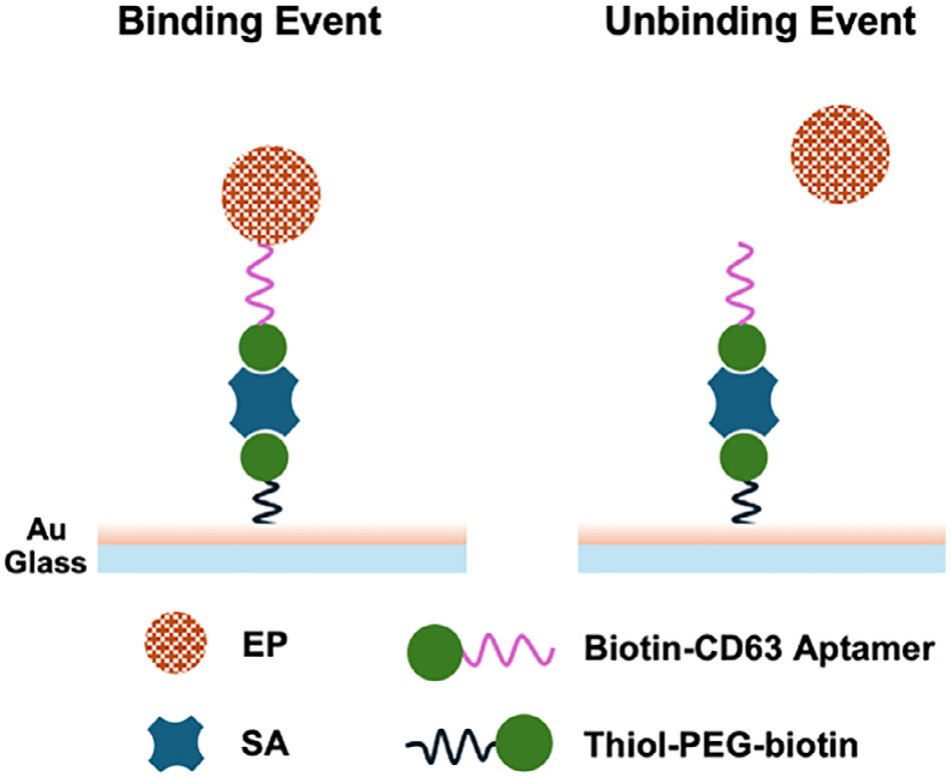


**FIGURE S9**. Experimental procedure for the interaction between EPs and CD63 aptamer, including the corresponding binding and unbinding events.

These corrections do not affect the overall findings and conclusions of the article.

We sincerely apologize for these errors.

