# Supplementary material for: Precise Sizing and Collision Detection of Functional Nanoparticles by Deep Learning Empowered Plasmonic Microscopy
Source: Adv Sci (Weinh). 2025 Jan 10;12(9):2407432. doi: 10.1002/advs.202407432 (PMC11884610; doi:10.1002/advs.202407432)
Supplement: Supplementary file 1 — Supporting Information [file ADVS-12-2407432-s002.docx]

Supporting Information

Precise sizing and collision detection of functional nanoparticles by deep learning empowered plasmonic microscopy

Jingan Wang^#^, Yi Sun^#^, Yuting Yang*, Cheng Zhang, Weiqiang Zheng, Chen Wang, Wei Zhang, Lianqun Zhou*, Hui Yu*, Jinghong Li*

**Supplementary Note 1. Shot noise removal principles**

The shot noise in surface plasmon resonance microscopy (SPRM) image sequence follows a spatial and temporal process which can be represented by the cox process. The Cox process is also known as the doubly stochastic Poisson process. Its first randomness refers to: when given an intensity 𝜦, the intensity of the Poisson process is a homogeneous Poisson process with 𝜦 as the intensity, which is in accord with the shot noise in SPRM image sequence caused by photon fluctuation of incident light (**Figure S1**). The second randomness refers to the fact that the intensity 𝜦 of the process is a random process, which corresponds to the intensity variation in the spatial space of SPRM image sequences.

${{\lambda\left( u,t \right)=\lambda}_{1}\left( u \right)\lambda}_{2}\left( t \right)S\left( u,t \right), ES\left( u,t \right)=1, \left( u,t \right)\epsilon\mathbb{R}^{2}\mathbb{\times Z}$ (1)

Where $\lambda_{1}\left( u \right)$ can be assumed to be the intensity variation in the spatial observation of SPRM sequences, and $\lambda_{2}\left( t \right)$ refers to the shot noise variation that follows the Poisson distribution over time. $S\left( u,t \right)$ represents the spatio-temporal process with unit mean [1].

The likelihood function $l\left( \theta;x \right)$of a Cox process of$X_{W\times T}$ can be expressed as,

$l\left( \theta;x \right)=\left[ \prod_{u\in X_{W}} \lambda_{1}\left( u \right) \right]\left[ \prod_{t\in T} \lambda_{2}\left( t \right)^{n_{t}} \right]\times E_{\theta}\left[ exp\left( -\sum_{t\in T} \int_{W} \lambda_{1}\left( u \right)\lambda_{2}\left( t \right)S\left( u,t \right)du \right)\prod_{t\in T} \prod_{u\in x_{t}} S\left( u,t \right) \right]$(2)

Where, $W\subset\mathbb{R}^{2}$ denotes an observed window of a finite point process $x_{t}\subset W$, $T\subset\mathbb{Z}$ represents a finite temporal observation window for each time t, and $n_{t}$ is the number of points observed at time t. It suggested that the unknown parameters ($\theta$) can be deduced with the covariates $S\left( u,t \right)$ between $\lambda_{1}\left( u \right)$ and $\lambda_{2}\left( t \right)$, emphasizing the importance of exploring the spatio-temporal correlation of the shot noise in increasing the sensitivity of SPRM.

**Supplementary Note 2. Structure of Deep-SM model**

The ResUNet structure in Deep-SM shown in **Figure. S2** adopts a 3$\times$3 convolution kernel with sliding step size of 1, to capture more detailed features. Batch Normalization (BN) Layer is added to each residual convolution module to make the scale of the input SPRM image sequence consistent (**Table S1**). It can reduce the difference between different batches and improve the robustness of the network and accelerate the convergence rate of network training. In addition, Linear Rectification (ReLU) layer is added after BN layer to better fit the nonlinear characteristics of the data and avoid the rapid decay of the network model gradient. In the down-sampling section of ResUNet, we adopted max pooling to increase the receptive field of SPRM image while reducing parameters and removing redundant information. The size of the convolution kernel used is 2$\times$2 to ensure that no excessive information is lost during down-sampling. During the up-sampling process, transposed convolution with convolution kernel 2$\times$2 was used to ensure the network outputs having the same size with the input image sequences which have high resolution. The number of channels in each layer in ResUNet is 64, 128, 256, 512, and 1024, respectively.

The Bi-ConvLSTM structure in Deep-SM is applied to better explore the temporal continuity of signals and spatiotemporal correlation of shot noise in SPRM sequences, we combined Bi-directional convolutional long short-term memory (Bi-ConvLSTM) network with Residual U-Net (ResUNet) to form Deep-SM. Bi-ConvLSTM is adapted from the convolutional LSTM, which overcomes the problem of gradient disappearing and gradient explosion that may occur when updating parameters. ConvLSTM model consists of three main gates to control the flow of information by gating mechanism, including forget gate, input gate and output gate [2]. The output of forget gate ($f_{t}$) determines which part of information in the memory cell of the previous time step ($C_{t-1}$) should be discarded.

$f_{t}=\sigma\left( W_{xf}*\mathcal{X}_{t}+W_{hf}*\mathcal{H}_{t-1}+b_{f} \right)$ (3)

The output of input gate ($i_{t}$) determines which part of information in the memory cell ($C_{t}$) should be updated.

$i_{t}=\sigma\left( W_{xi}*\mathcal{X}_{t}+W_{hi}*\mathcal{H}_{t-1}+b_{i} \right)$ (4)

$\tilde{\mathcal{C}_{t}}=\tanh\left( W_{xc}*\mathcal{X}_{t}+W_{hc}*\mathcal{H}_{t-1}+b_{c} \right)$ (5)

$\mathcal{C}_{t}=f_{t}⨀\mathcal{C}_{t-1}+i_{t}⨀\tilde{\mathcal{C}_{t}}$ (6)

The output of output gate ($o_{t}$) controls how much $C_{t}$ should influence the output of the hidden layer at the current time step ($H_{t}$). The formular of ConvLSTM() is as follows,

$o_{t}=\sigma\left( W_{xo}*\mathcal{X}_{t}+W_{ho}*\mathcal{H}_{t-1}+b_{o} \right)$ (7)

$\mathcal{H}_{t}=o_{t}\tanh\left( \mathcal{C}_{t} \right)$ (8)

Where $\mathcal{X}_{t}$ is the input vector to ConvLSTM at time $t$, $W$ and $b$are weight matrices and bias vector parameters for three gates. $\sigma$ and $\tanh$ refer to sigmoid and hyperbolic tangent activation function. $*$ denotes convolution operation, and $⨀$ represents the Hadamard product (element-wise product).

Although ConvLSTM can fully capture the spatiotemporal information of signals and shot noise, it can only transmit information from front to back on the timeline of SPRM image sequences. Bi-ConvLSTM in Deep-SM which consists of two separate ConvLSTM modules with the input SPRM sequence flows in both directions can effectively solve this problem. The hidden layer state of the Bi ConvLSTM network is obtained by combining the forward LSTM hidden layer state ($\vec{\mathcal{H}_{t}}$) and the reverse LSTM hidden layer state ($\mathcal{H}_{t}$).

$\vec{\mathcal{H}_{t}}=CONVLSTM\left( \mathcal{H}_{t-1},\mathcal{X}_{t} \right)$ (9)

$\mathcal{H}_{t}=CONVLSTM\left( \mathcal{H}_{t+1},\mathcal{X}_{t} \right)$ (10)

$\mathcal{H}_{t}=\alpha\vec{\mathcal{H}_{t}}+\beta\mathcal{H}_{t}$ (11)

**Supplementary Note 3. SNR definition**

The definition of signal-to-noise ratio (SNR) is based on the following formula, where the signal here refers to the brightest point of the GNP signal.

$SNR=20\log_{10} \left( \frac{Signal-mean(Background Noise)}{std(Background Noise)} \right)$ (12)

**Supplementary Note 4. Interferometric reconstructed procedure**

The interferometric reconstructed process shown in **Figure S7** was based on filtered deconvolution of SPRM image sequence. We first transferred the SPRM images to Fourier Domain by Fourier Transform (FT), and then applied a low pass filter to remove the overlapping part of the conjugate rings in the frequency domain. Then, we transferred them back to the image domain by Inverse Fourier Transform (IFT) and then multiplied the incident light field. After applying the ring filter in the frequency domain and back to the image domain again, we apply iterative-based Richardson-Lucy (RL) deconvolution to achieve the final reconstructed dotlike result ($O$). The iteration number of RL deconvolution ($k$) was set to 50, and it could be adjusted based on the image quality.

$O^{k+1}=\left[ \frac{DeepSM Output}{psf*O^{k}}*{psf}^{T} \right]O^{k}$ (13)

The point spread function ($psf$) used in the deconvolution was a SPRM image of a 50 nm gold nanoparticle (GNP) with ultra-high SNR. The intensity of nanoparticles in the reconstructed results was calculated by the average in 5$\times$5 area around the brightest points. Here, we show the reconstruction results of both four average (FA) and Deep-SM results with four input SPRM frames of an extracellular particle (EP) from PANC-1 human pancreatic cancer cell line. We succeeded in localizing the EPs in the reconstructed result of Deep-SM result while failed in FA.

**Supplementary Note 5. Determination of EP Size**

To determine the size of extracellular vesicles (EPs), we first established the calibration curve correlating the intensity in the reconstructed plasmonic microscopy images with particle sizes. This was done using polystyrene nanoparticles (PSNPs) as size standards. In this manuscript, we utilized PSNPs with diameters of 20 nm, 30 nm, 50 nm, 100 nm and 150 nm to construct the size calibration curves (**Figure S8**). Since the different nanoparticles have different dielectric constants and different refractive index, we calculated the size relationship between EPs and PSNPs based on their scattering cross section ($\sigma$) in plasmonic microscopy [3].

$\alpha=3V\left( n_{s}^{2}-n_{m}^{2} \right)\left( n_{s}^{2}+2n_{m}^{2} \right)^{-1}$ (14)

$\sigma= \frac{8}{3}\pi^{3}\alpha^{2}{(\frac{\lambda}{n_{m}})}^{-4}$ (15)

Where V is the volume of the nanoparticles, $\alpha$ denotes the polarizability of the incident electric field, $n_{s}$ and $n_{m}$ represent the refractive index of the nanoparticles and surrounding medium, respectively, and $\lambda$ is the wavelength of the incident light. $n_{s}$ of EPs is ~1.39 and $n_{s}$of PSNPs is ~1.55 [4]. Therefore, the size of EPs can be deduced from their relationship with PSNPs. We have supplemented this content in the Supporting Information.

$\sigma_{PSNP}\sim\left( \left( \frac{R_{PSNP}}{2} \right)^{3}\left( {1.55}^{2}-{1.33}^{2} \right)\left( {1.55}^{2}+2{\times1.33}^{2} \right)^{-1} \right)^{2}\approx1.778e^{-4}{R_{PSNP}}^{6}$ (16)

$\sigma_{EP}\sim\left( \left( \frac{R_{EP}}{2} \right)^{3}\left( {1.39}^{2}-{1.33}^{2} \right)\left( {1.39}^{2}+2{\times1.33}^{2} \right)^{-1} \right)^{2}\approx1.391e^{-5}{R_{EP}}^{6}$ (17)

$R_{EP}=\left( \frac{1.778e^{-4}}{1.391e^{-5}} \right)^{1/6}\approx1.529\times R_{PSNP}$ (18)

**Supplementary Note 6. Specific and Non-specific Binding Events of EPs.**

The interaction between EPs and specifically modified surfaces (such as CD63 aptamers) can be either specific or non-specific, directly influencing the binding time or bound lifetime of EPs on the Au chip surface. The distinction between specific and non-specific binding is primarily determined by analyzing the binding time. A method that differentiates specific and non-specific binding has been previously reported in our earlier publications [5]. The bound lifetime of EPs, including both specific and non-specific binding, can be effectively modeled using the exponential function provided below:

$y=y_{0}+A_{0}e^{-\frac{t}{\tau}}, y_{0}>0$ (19)

where $\tau$ represents the mean bound lifetime. We analyzed the bound lifetime of 500 EPs with scrambled aptamer (control group) and 1200 EPs with CD63 aptamer. Non-specific binding events typically exhibit Brownian motion and generally have a bound lifetime of less than 1 s. The mean lifetime of EPs with scramble aptamer is 2.51$\pm$0.08 s, and 20.69$\pm$1.58 s for the CD63 aptamer. A threshold value, calculated as the mean bound lifetime in the control group plus three times the SD of the mean, was applied to distinguish between non-specific binding and specific binding events of EPs.

**Supplementary Note 7. Phase Quantification Metric**

We quantified the phase change of single nanoparticles during the collision and electrochemistry process by structure similarity index measure (SSIM). SSIM evaluates the perceived quality of SPRM images from three aspects: luminance, contrast, and structure,

$SSIM\left( I_{E},I_{S} \right)=\frac{\left( 2\mu_{E}\mu_{s}+\left( K_{1}L \right)^{2} \right)\left( 2\sigma_{ES}+\left( K_{2}L \right)^{2} \right)}{\left( {\mu_{E}}^{2}+{\mu_{S}}^{2}+\left( K_{1}L \right)^{2} \right)\left( {\sigma_{E}}^{2}+{\sigma_{S}}^{2}+\left( K_{2}L \right)^{2} \right)}$ (20)

where $\mu_{E}$, $\mu_{S}$, $\sigma_{E}$, $\sigma_{S}$ represent the mean value and variance of $I_{E}$ and $I_{S}$, respectively. $\mu_{ES}$ is the covariance between $I_{E}$ and $I_{S}$, and $L$ is the dynamic range of the SPRM images ([0, 255]). Here, we set $K_{1}$ to 0.01 and $K_{2}$ to 0.03.

$\mu_{E}=\frac{1}{N}\sum_{i=1}^{N} I_{E}, \mu_{S}=\frac{1}{N}\sum_{i=1}^{N} I_{S}$ (21)

$\mu_{ES}=\frac{1}{N-1}\sum_{i=1}^{N} \left( {I_{E}}_{i}-\mu_{E} \right)\left( {I_{S}}_{i}-\mu_{S} \right)$ (22)

$\sigma_{E}=\frac{1}{N-1}\sqrt{\sum_{i=1}^{N} {({I_{E}}_{i}-\mu_{E})}^{2}}, \sigma_{S}=\frac{1}{N-1}\sqrt{\sum_{i=1}^{N} {({I_{S}}_{i}-\mu_{S})}^{2}}$ (23)

**Supplementary Note 8. Determination of PEG length.**

The maximal displacement of nanoparticles depends on two factors: the diameter of the nanoparticles and length of tethered PEG length [6]. The PEG length in its natural and unperturbed state is determined by its molecular formular, HO(CH2CH2O)_n_H, where n refers to the number of ethylene glycol monomer units (CH2CH2O), which corresponds to the degree of polymerization. The parameter n can be calculated by the following equation:

$n= \frac{PEG Molecular Weight}{atomic weight of O\times2+atomic weight of H\times4+atomic weight of C\times1}$ (24)

Therefore, the PEG length in its natural and unperturbed state is approximately 21.63nm for PEG chains with molecular weight of 3400 Da and 63.56 nm for PEG chains with molecular weight of 10000 Da [7]. The PEG chains we selected were flexible enough which allows for significant degrees of extension. As the accuracy in x- and y- localization is low for large z-value, we focused on those data with z < 20 nm and analysed the maximum x- and y- motion. This is reasonable as the maximized x and y location should be observed at z=0. The updated value corresponds to ~ 91% of the full length of PEG-linker.

Figure S1.


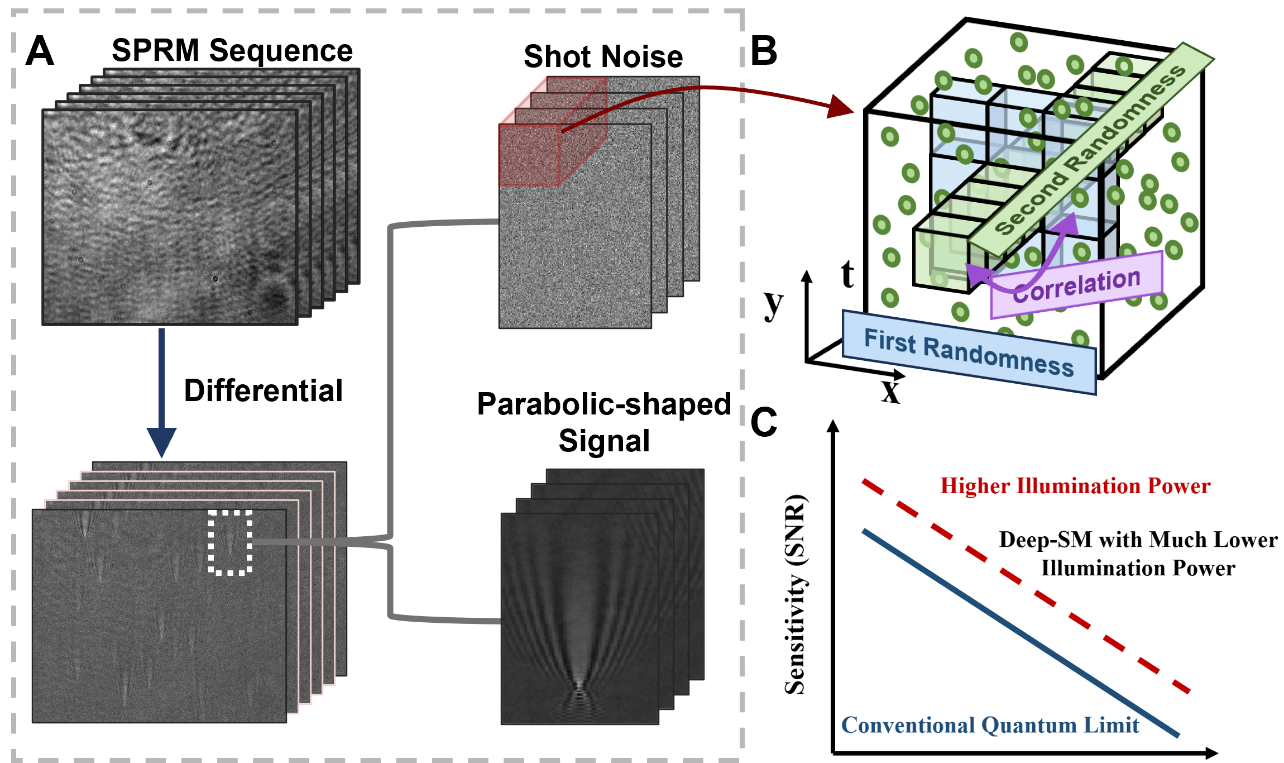


**Figure S1.** Generation mechanism of shot noise in SPRM image sequences and capability of Deep-SM. (A) The differential SPRM image sequences acquired by subtracting the statistic background from the raw image sequences were composed of both parabolic-shaped signal and shot noise. (B) The shot noise could be expressed as two-dimensional randomness based on the Cox process. (C) Deep-SM could improve the sensitivity of SPRM with lower illumination power by exploring the spatio-temporal correlation of shot noise and the spatial features of the signal.

Figure S2.


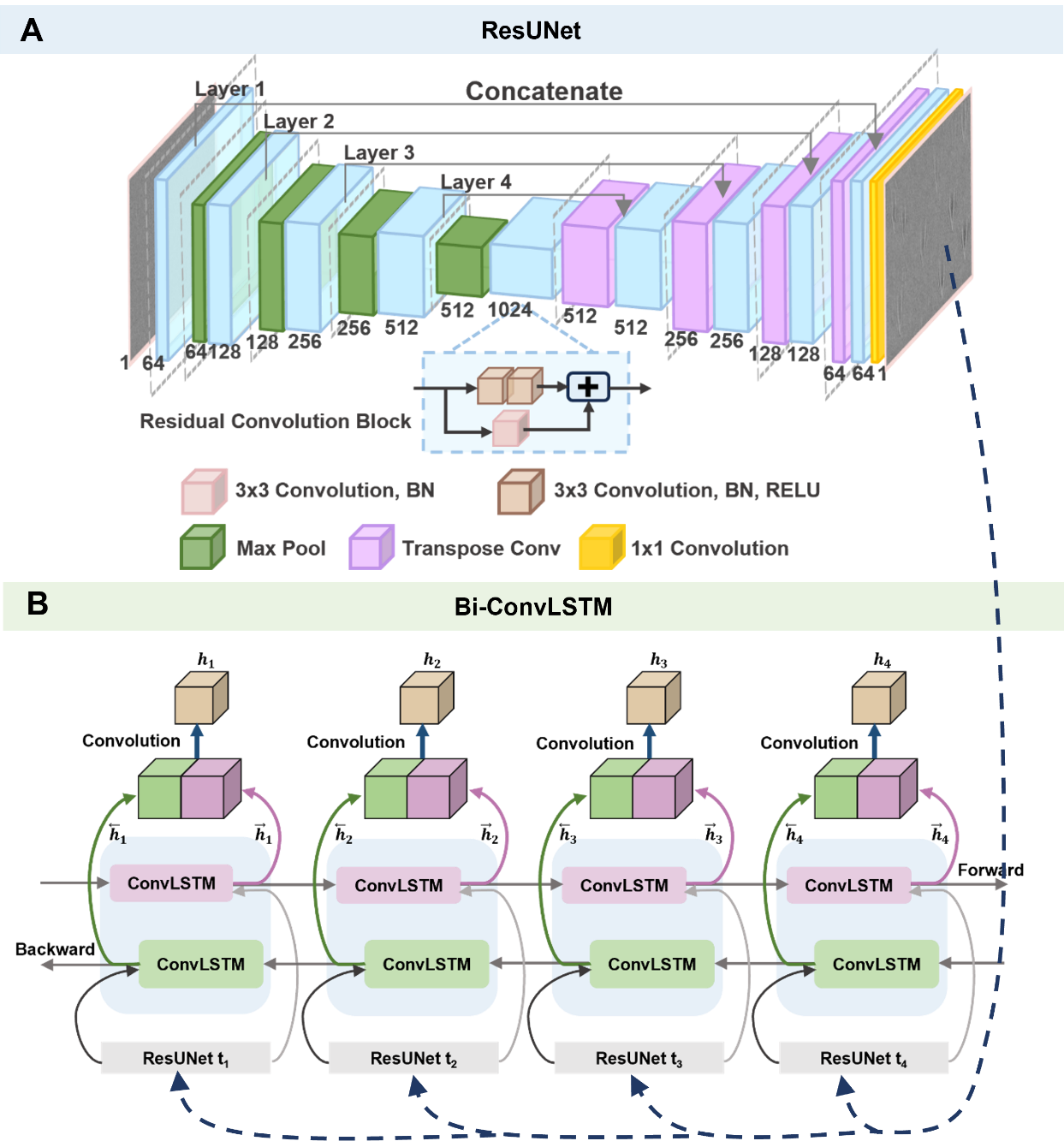


Figure S2. Deep-SM is composed of a series of ResUNet and Bi-ConvLSTM networks. (A) In the structure of ResUNet in Deep-SM, the double convolution block in traditional U-Net is replaced by the residual convolution block in Deep-SM structure. (B) The structure of Bi-directional convolutional long short-term memory (Bi-ConvLSTM) network in Deep-SM.

Figure S3.


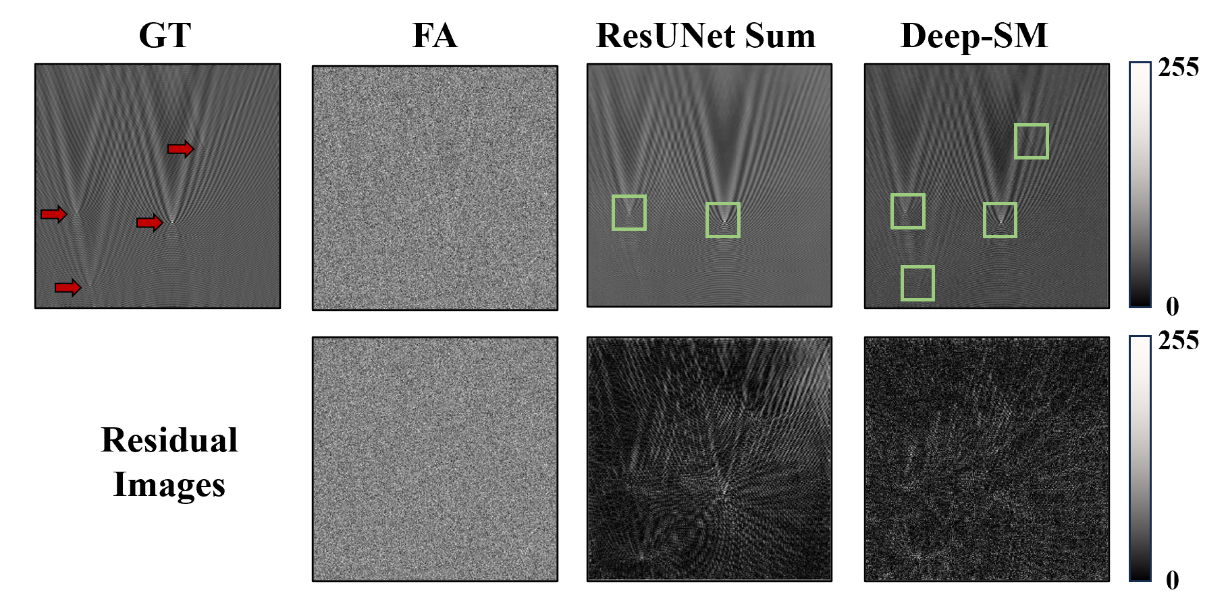


Figure S3. Deep-SM performance on simulated SPRM images with Poisson noise. Four nanoparticles with different intensities were simulated in the SPRM sequence, and artificial Poisson noise was added to the simulated images. The residual images calculated between the test results and the Ground Truth (GT) turned out that Deep-SM could enhance the detection sensitivity significantly and enable weak signal detection. In contrast, we failed to observe the nanoparticles with low intensity in FA detection and the sum of the ResUNet outputs.

Figure S4.


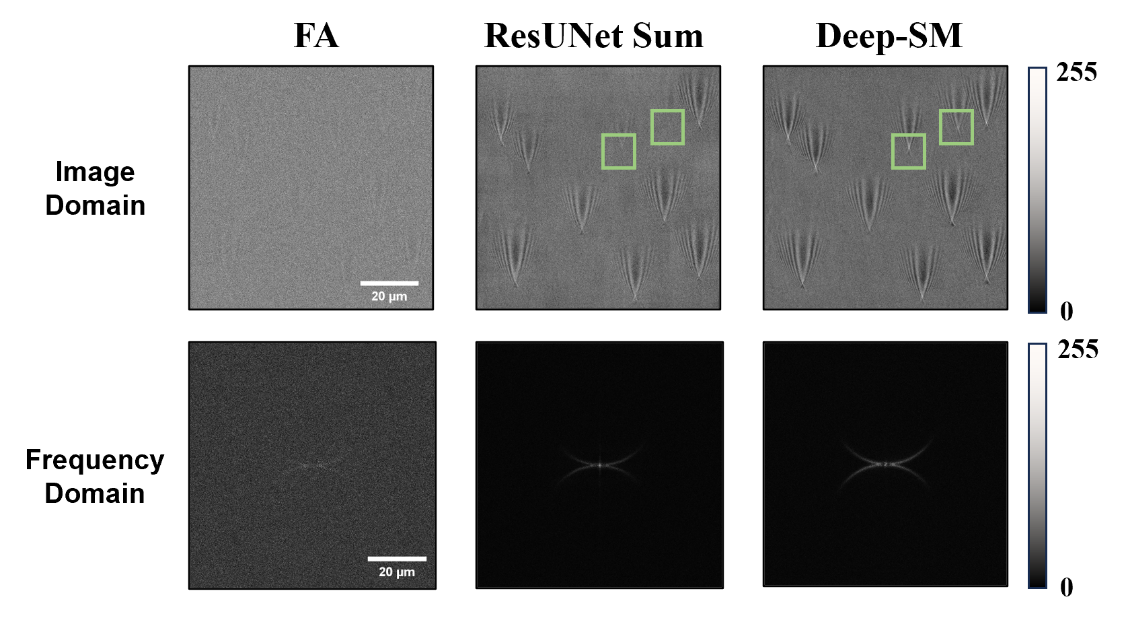


Figure S4. Deep-SM test on experimental SPRM images of 5 nm GNPs and frequency analysis. GT, FA, ResUNet sum result, Deep-SM test result of 5 nm GNPs, and their corresponding spectrum in the frequency domain.

Figure S5.


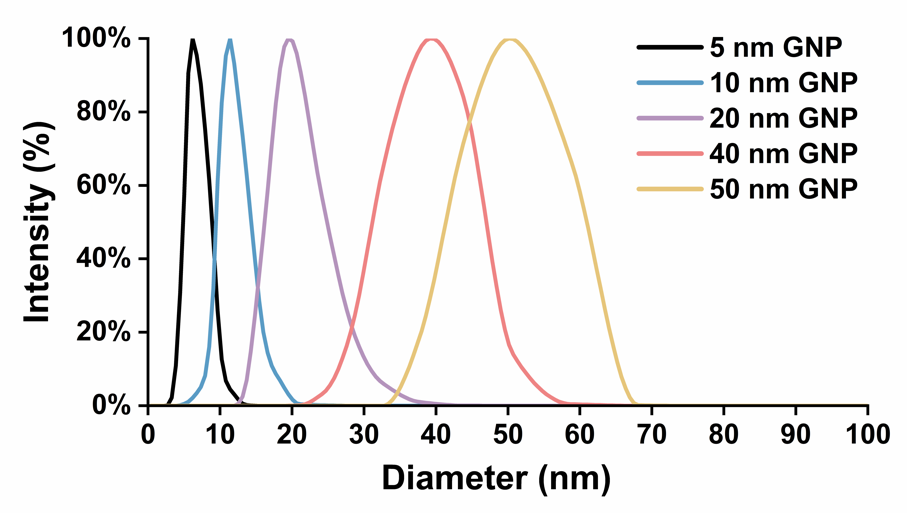


Figure S5. Dynamic light scattering (DLS) measurements for 5 nm, 10 nm, 20 nm, 40 nm, 50 nm GNPs. The output of DLS measurements represents the normalized distribution.

Figure S6.


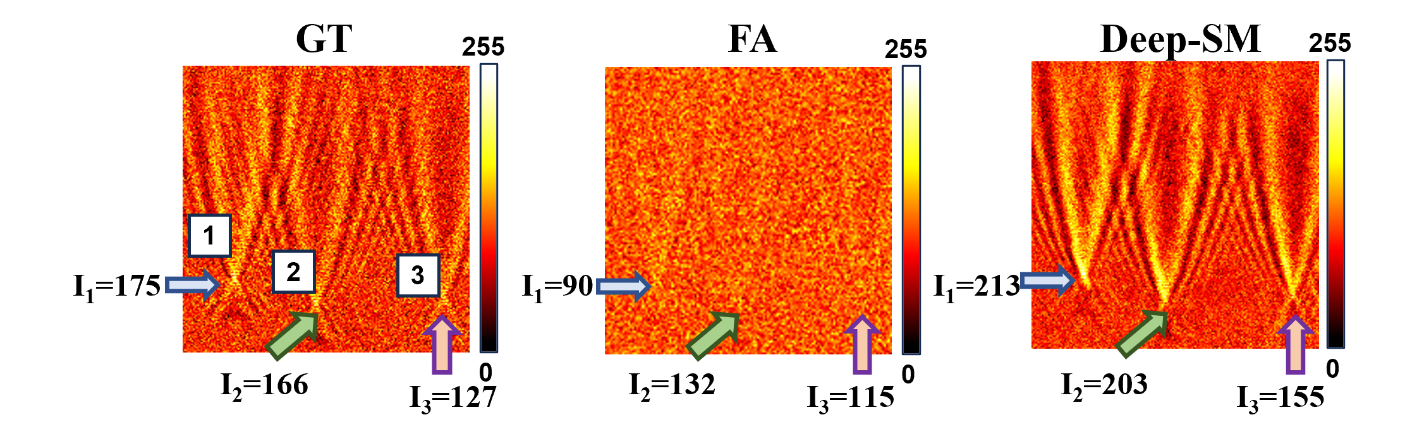


Figure S6. Intensity Comparison of GT, FA, Deep-SM Results of 5 nm GNPs with the same SPRM image sequence. The intensity of 5 nm GNPs detected in the GT was proportional to the intensity detected by Deep-SM. The ratio of the intensity detected by Deep-SM results and GT was ~ 1.22 (213/175$\boldsymbol{\approx}$1.2171, 203/166$\boldsymbol{\approx}$1.2229 155/127$\boldsymbol{\approx}$1.2205) for all these three particles, which demonstrated the enhancement consistency and stability of Deep-SM on particles with different intensity. In contrast, the intensity of particles detected in FA was inconsistent with the GT results (90/175$\boldsymbol{\approx}$0.5143, 132/166$\boldsymbol{\approx}$0.7952, 115/127$\boldsymbol{\approx}$0.9055).

Figure S7.


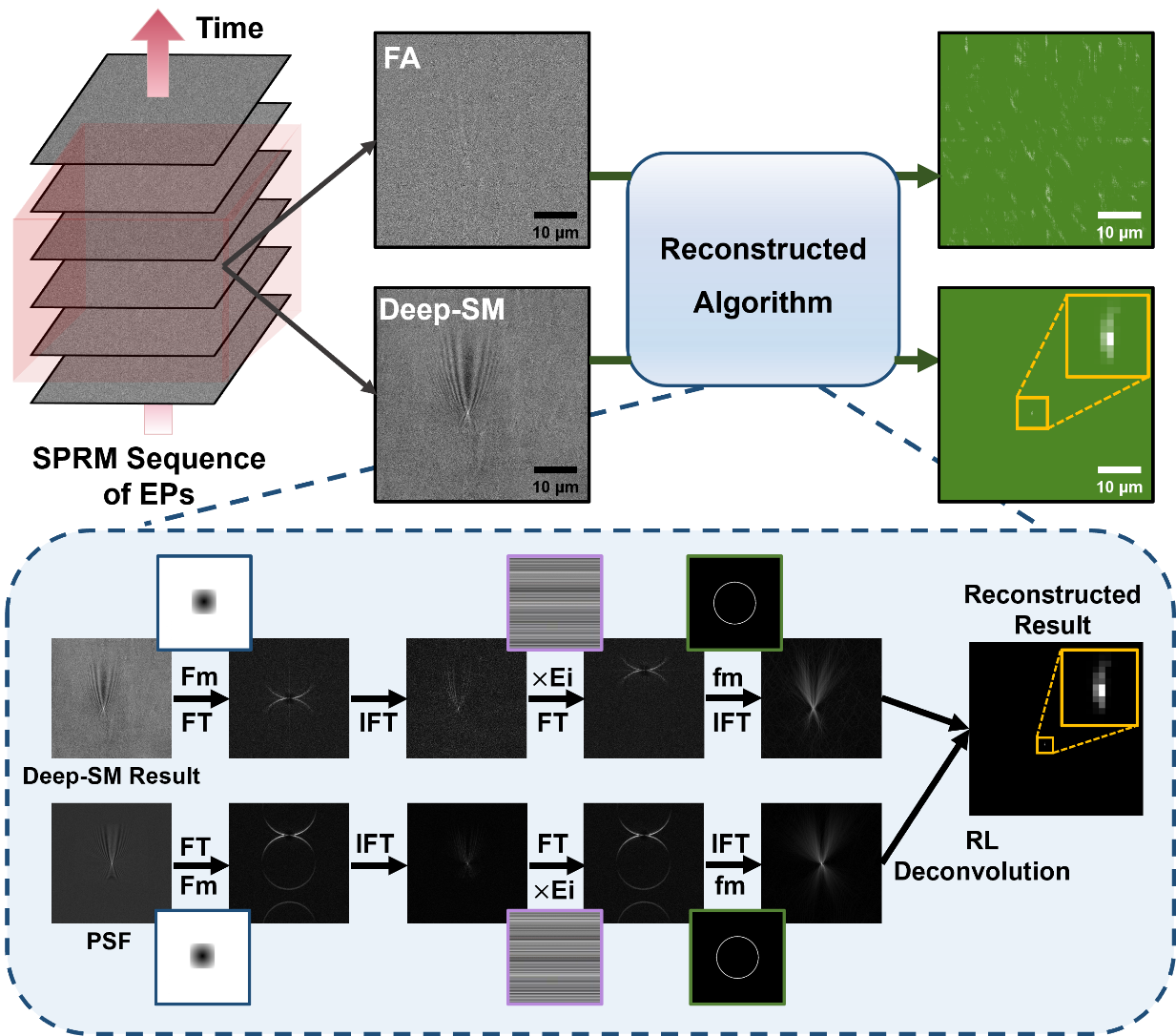


Figure S7. The interferometric reconstruction procedure and results of extracellular particles (EPs). The EPs could be detected directly in the reconstructed results of Deep-SM, while failed in FA reconstructed results.

Figure S8.


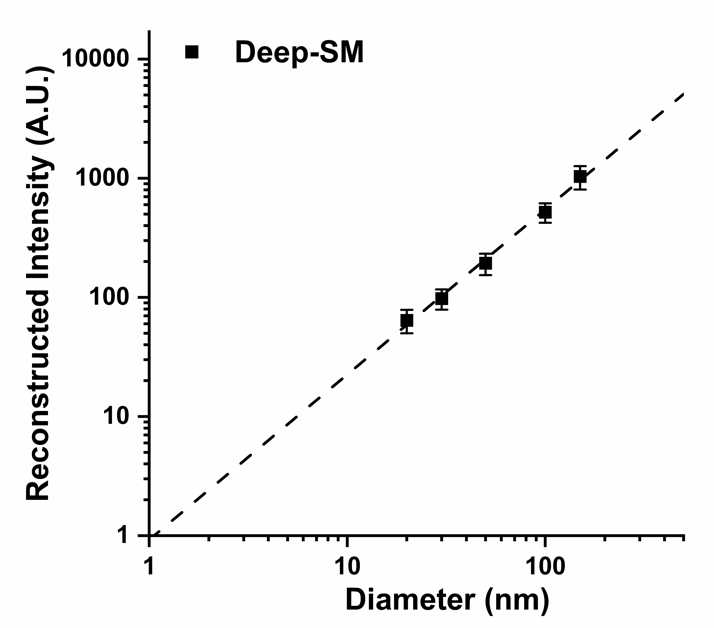


**Figure S8.** Calibration curve of PSNPs with diameters of 20 nm, 30 nm, 50 nm, 100 nm and 150 nm.

Figure S9.


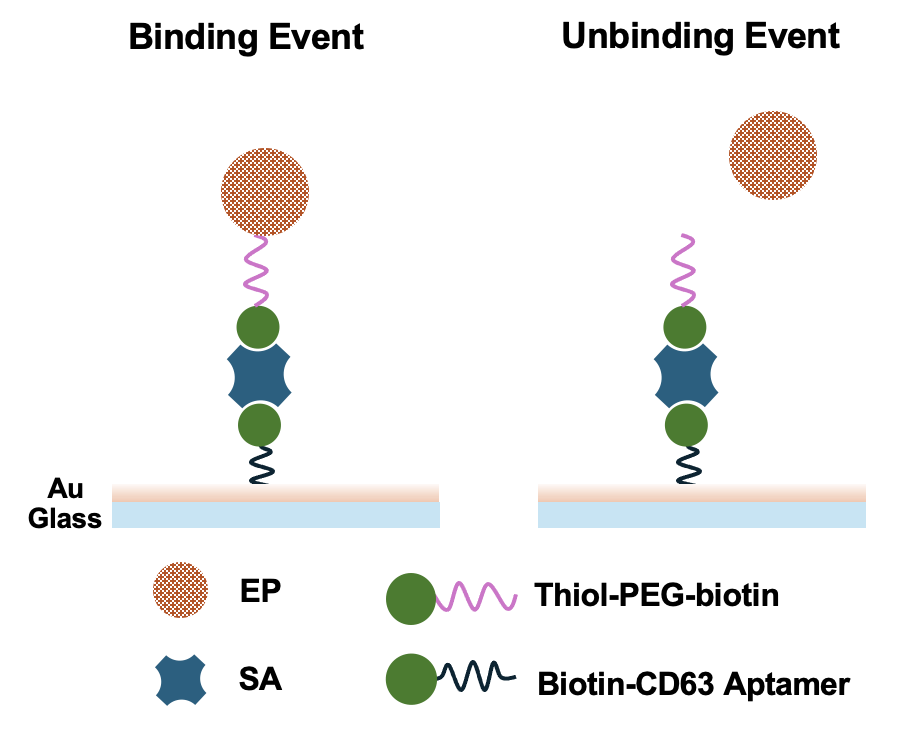


**Figure S9.** Experimental procedure for the interaction between EPs and CD63 aptamer, including the corresponding binding and unbinding events.

Figure S10.


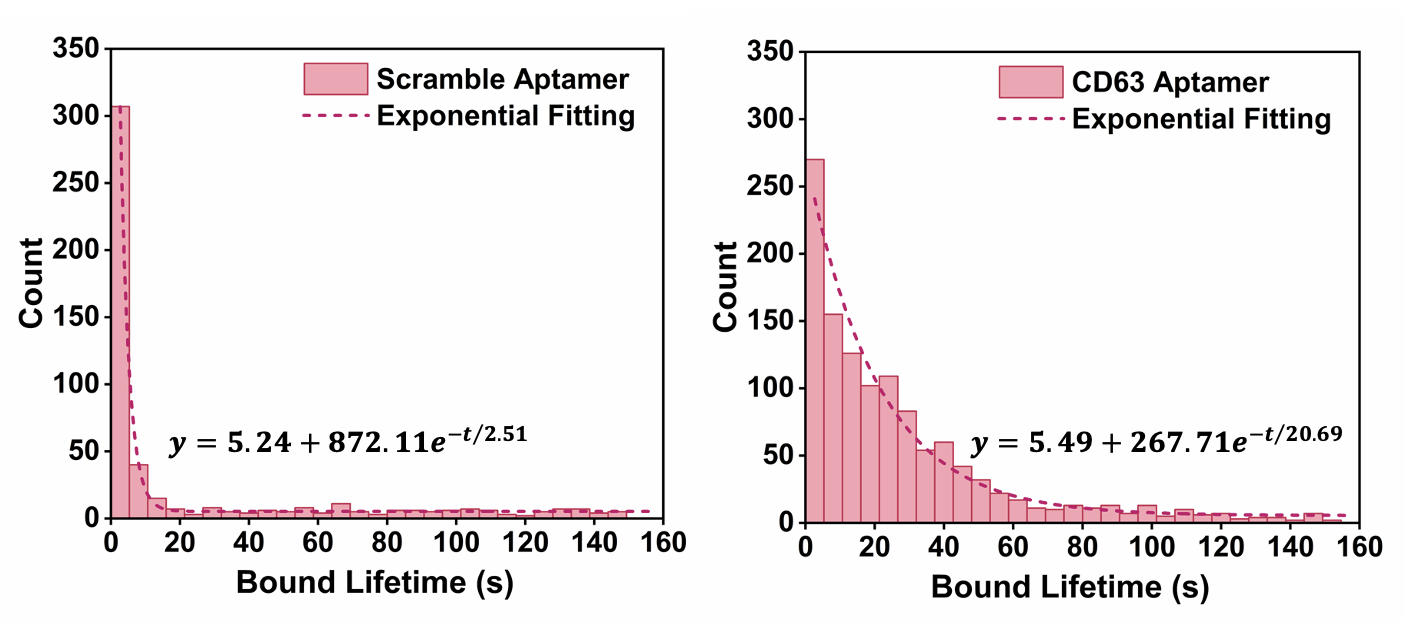


**Figure S10.** Bound lifetime distribution and exponential fitting results of EPs with CD63 aptamer and scrambled aptamer.

Figure S11.


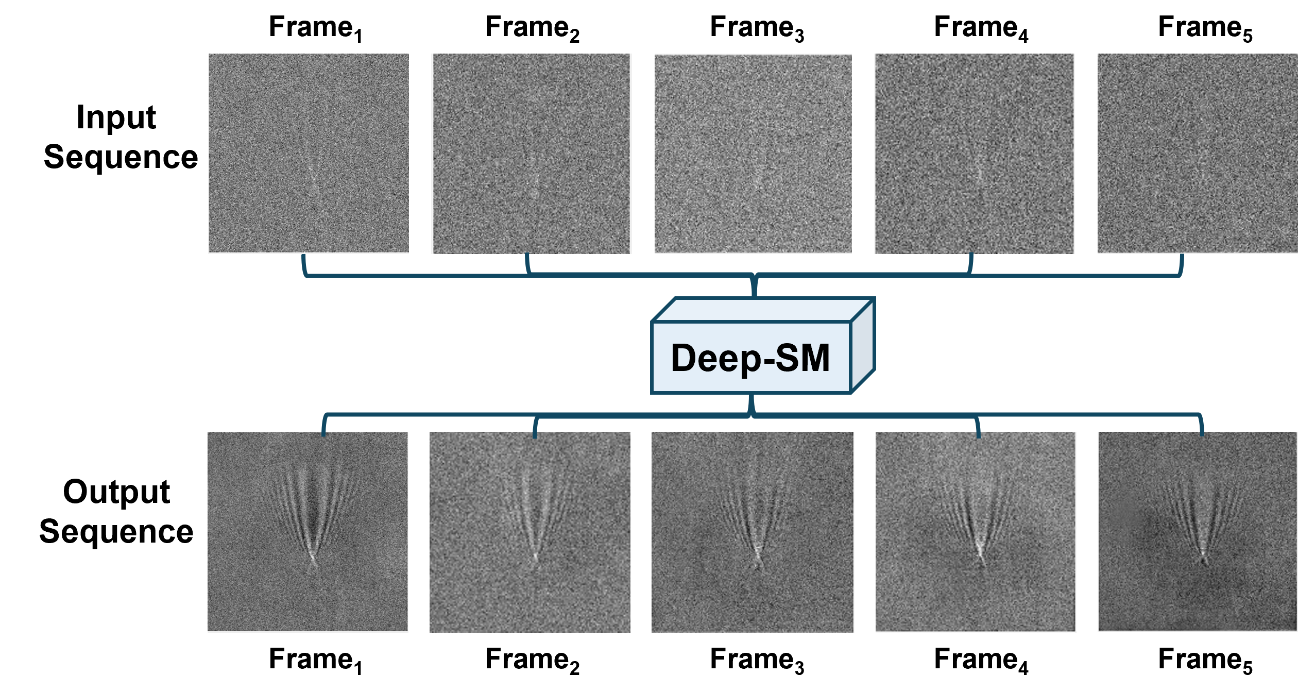


Figure S11. Phase detection with Deep-SM with high temporal resolution. To prove the ability of Deep-SM on SPRM image sequences with rapid phase changing, we chose five SPRM frames changed to white-center (phase$\boldsymbol{\approx}$0) from black-center (phase$\boldsymbol{\approx\pi}$) as the Deep-SM inputs. It turned out that Deep-SM worked efficiently in rapid phase change detection as well, while it might scarify some sensitivity compared with the smooth phase change detection.

Figure S12.


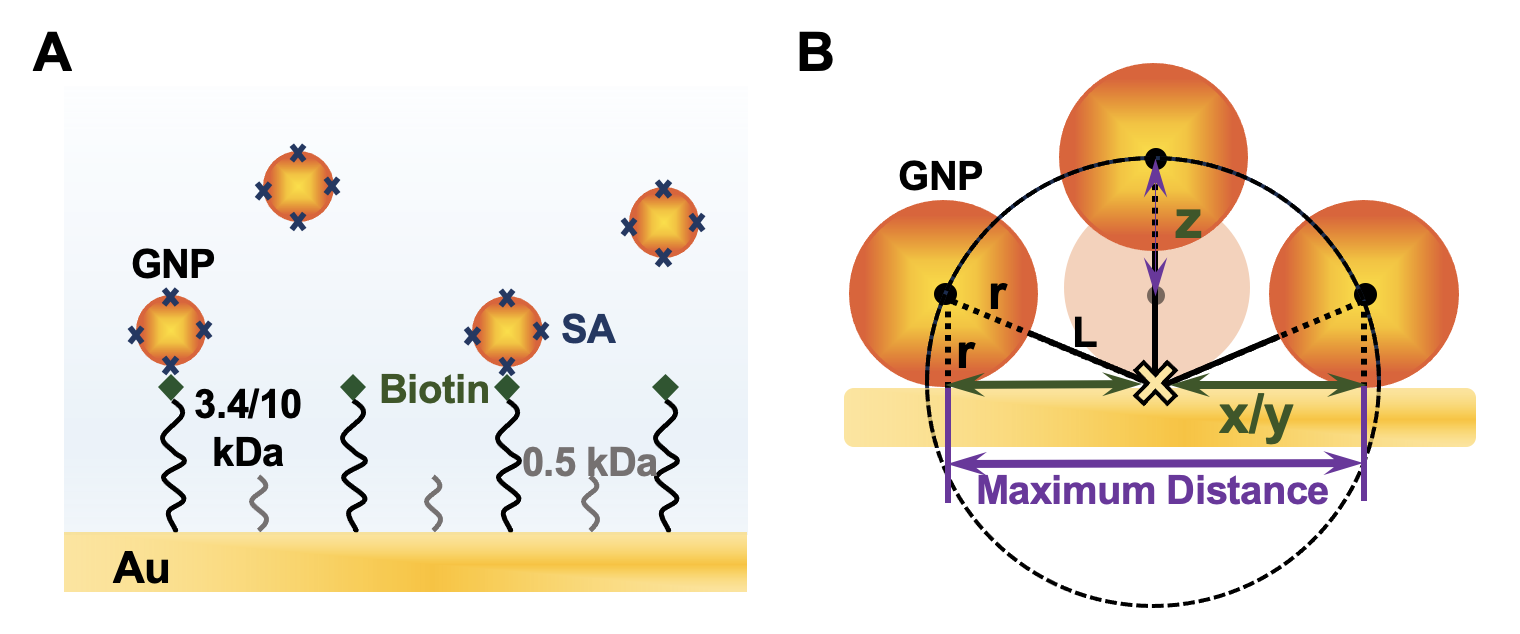


Figure S12. Schematic of GNP Motion tracking with different PEG Chain Length. (A) The mixture of m-PEG-SH chains with molecular weight 500 Da and biotin-PEG-SH (molecular weight: 3.4 kDa or 10 kDa) chains were added to polydimethylsiloxane (PDMS) on Au film, respectively. After incubating overnight, we then replaced the liquid in PDMS by $\boldsymbol{\times}$1 pbs and 50 nm biotin-coated GNPs to observe the motion of GNPs. (B) The trajectory of GNP centers should be in the sphere, with the position of the peg chain bottom as the center, and the sum of GNP radius (r) and PEG Length (L) as the radius. The moving distance of GNPs in X, Y, and Z directions was calculated by the position of GNP centers. The maximum distance was calculated by the difference between the farthest positions from the center.

Figure S13.


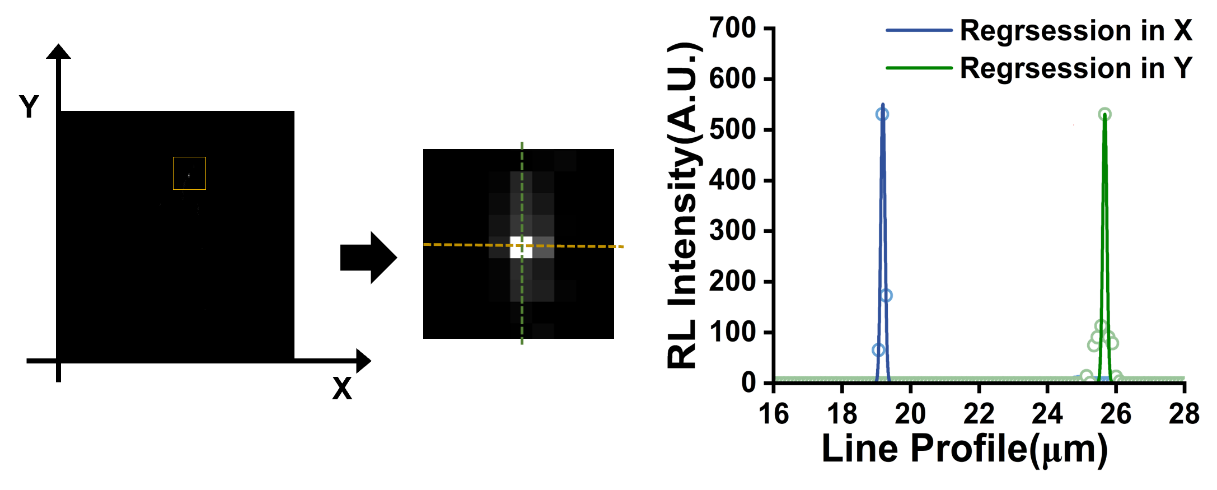


Figure S13. Localization of nanoparticles in the interferometric reconstruction results. The localization of the nanoparticles was determined by the maximum of intensity variation fitted by Gaussian distribution in X and Y directions.

Figure S14.


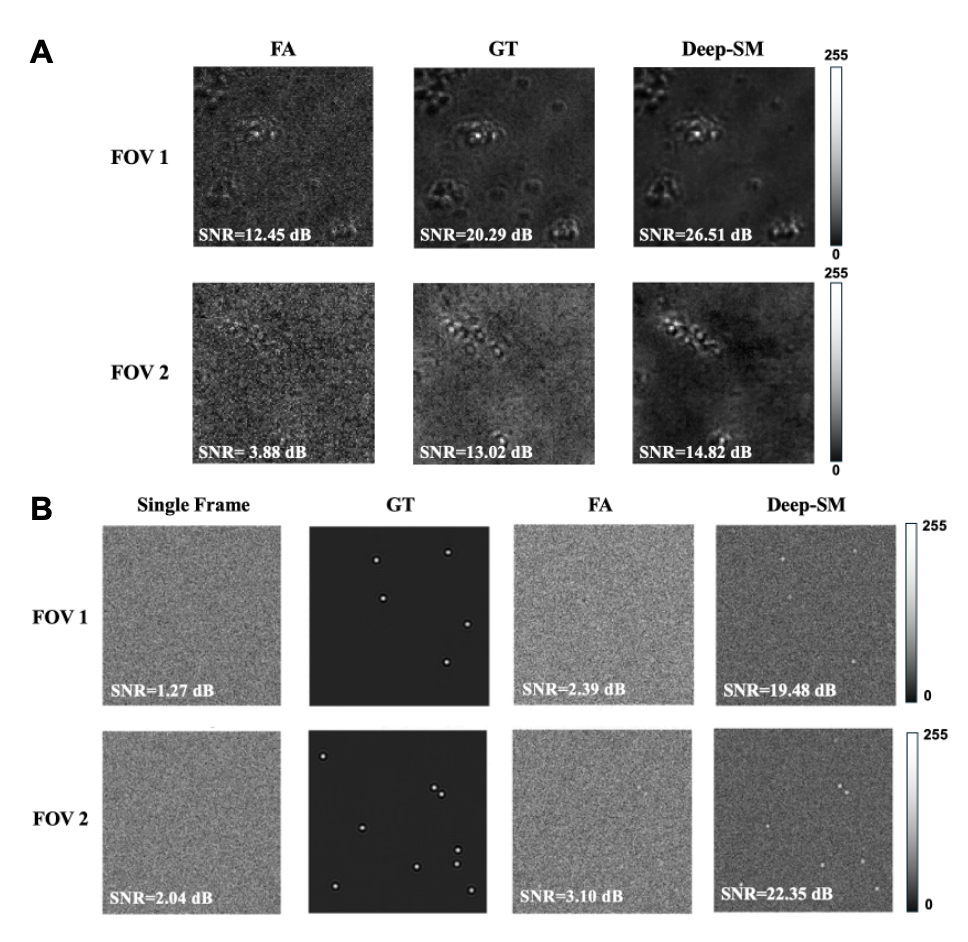


**Figure S14.** Evaluation of Deep-SM on experimental dark-field microscopy (DFM) and simulated iSCAT image sequences. (A) Evaluation of Deep-SM on experimental DFM image sequences obtained from 50 nm GNPs. Using Deep-SM, the nanoparticles could be easily distinguished from the background. (B) The iSCAT image sequences were simulated with the addition of Poisson noise, Deep-SM could improve the SNR of iSCAT images dramatically.

Table S1. Specific structure and parameters of ResUNet in Deep-SM.

|  | **Depth** | **Layer** | **Filter/Channel** | **Stride** | **Output Size** |
| --- | --- | --- | --- | --- | --- |
| **Input** |  |  | 3×3 / 1 |  | W×H×1 |
| **Encoding Section** | 1 | Conv + BN + ReLU | 3×3 / 64 | 1 | W×H×64 |
|  |  | Conv + BN + ReLU | 3×3 / 64 | 1 | W×H×64 |
|  |  | Addition |  |  | W×H×64 |
|  |  | Max Pooling | 2×2 / 64 | 2 | W/2×H/2×64 |
|  | 2 | Conv + BN + ReLU | 3×3 / 128 | 1 | W/2×H/2×128 |
|  |  | Conv + BN + ReLU | 3×3 / 128 | 1 | W/2×H/2×128 |
|  |  | Addition |  |  | W/2×H/2×128 |
|  |  | Max Pooling | 2×2 / 128 | 2 | W/4×H/4×128 |
|  | 3 | Conv + BN + ReLU | 3×3 / 256 | 1 | W/4×H/4×256 |
|  |  | Conv + BN + ReLU | 3×3 / 256 | 1 | W/4×H/4×256 |
|  |  | Addition |  |  | W/4×H/4×256 |
|  |  | Max Pooling | 2×2 / 256 | 2 | W/8×H/8×256  单个 |
|  | 4 | Conv + BN + ReLU | 3×3 / 512 | 1 | W/8×H/8×512 |
|  |  | Conv + BN + ReLU | 3×3 / 512 | 1 | W/8×H/8×512  ]\ |
|  |  | Addition |  |  | W/8×H/8×512 |
|  |  | Max Pooling | 2×2 / 512 | 2 | W/16×H/16×512 |
| **Bridge** | 5 | Conv + BN + ReLU | 3×3 / 1024 | 1 | W/16×H/16×1024 |
|  |  | Conv + BN + ReLU | 3×3 / 1024 | 1 | W/16×H/16×1024 |
|  |  | Addition |  |  | W/16×H/16×1024 |
| **Decoding Section** |  | Transposed Conv | 2×2 / 512 | 2 | W/8×H/8×512 |
|  | 4 | Concatenation | /1024 |  | W/8×H/8×1024 |
|  |  | Conv + BN + ReLU | 3×3 / 512 | 1 | W/8×H/8×512 |
|  |  | Conv + BN + ReLU | 3×3 / 512 | 1 | W/8×H/8×512 |
|  |  | Addition |  |  | W/8×H/8×512 |
|  |  | Transposed Conv | 2×2 / 256 | 2 | W/4×H/4×256 |
|  | 3 | Concatenation | /512 |  | W/4×H/4×512 |
|  |  | Conv + BN + ReLU | 3×3 / 256 | 1 | W/4×H/4×256 |
|  |  | Conv + BN + ReLU | 3×3 / 256 | 1 | W/4×H/4×256 |
|  |  | Addition |  |  | W/4×H/4×256 |
|  |  | Transposed Conv  Convolution | 2×2 / 128 | 2 | W/2×H/2×128 |
|  | 2 | Conv | /256 |  | W/2×H/2×256 |
|  |  | Conv + BN + ReLU | 3×3 / 128 | 1 | W/2×H/2×128 |
|  |  | Conv + BN + ReLU | 3×3 / 128 | 1 | W/2×H/2×128 |
|  |  | Addition |  |  | W/2×H/2×128 |
|  |  | Transposed Conv | 2×2 / 64 | 2 | W×H×64 |
|  | 1 | Concatenation | /128 |  | W×H×128 |
|  |  | Conv+ BN + ReLU | 3×3 / 64 | 1 | W×H×64 |
|  |  | Conv+ BN + ReLU | 3×3 / 64 | 1 | W×H×64 |
|  |  | Addition |  |  | W×H×64 |

**Table S2. CD63 aptamer and scrambled aptamer**

| Aptamer | Sequence (5’$\boldsymbol{\to}$3’) | Affinity (nmol·L-1） |
| --- | --- | --- |
| CD63 | CAC CCC ACC TCG CTC CCG TGA CAC TAA TGC TAT TTT TTT TTT-biotin | 17.1 |
| Scrambled Aptamer | GCT ACC TCC CGA TAT TGA GGG CGC CCT CGT CTT TTT TTT TTT-biotin | / |

Movie S1.

The specific binding process of EPs detected by FA and Deep-SM shown in Fig. 3F.

Movie S2.

The compositional evolution process of a single Ag to AgCl nanoparticle detected by FA and Deep-SM shown in Fig. 4J and Fig. 4K.

References

[1] J. Møller, C. Díaz‐Avalos, *Scandinavian Journal of Statistics* **2010**, *37* (1), 2.

[2] F. Xu, H. Ma, J. Sun, R. Wu, X. Liu, Y. Kong, in *2019 IEEE 4th International Conference on Image, Vision and Computing (ICIVC)* **2019**, 236-240.

[3] X. Fan, W. Zheng, D. Singh, *Light: Science & Applications* **2014**, *3*, e179.

[4] a) C. Gardiner, M. Shaw, P. Hole, J. Smith, D. Tannetta, C. W. Redman, I. L. Sargent, *Journal of Extracellular Vesicles* **2014**, *3* (1), 25361; b) Y. Lu, Y. Yin, Z.-Y. Li, Y. Xia, *Langmuir* **2002**, *18* (20), 7722.

[5] Q. Zeng, X. Zhou, Y. Yang, Y. Sun, J. Wang, C. Zhai, J. Li, H. Yu, *Proceedings of the National Academy of Sciences* **2022**, *119* (10), e2120379119.

[6] G. Ma, Z. Wan, Y. Yang, W. Jing, S. Wang, *ACS Sensors* **2021**, *6* (11), 4234.

[7] G. Ma, G. D. Syu, X. Shan, B. Henson, S. Wang, P. J. Desai, H. Zhu, N. Tao, *J Am Chem Soc* **2018**, *140* (36), 11495.
